# A review of microsampling techniques and their social impact

**DOI:** 10.1007/s10544-019-0412-y

**Published:** 2019-08-15

**Authors:** Benson U. W. Lei, Tarl W. Prow

**Affiliations:** 1 Future Industries Institute, University of South Australia, Mawson Lakes Campus, Building MM – MM2-01F, GPO Box 2471, Mawson Lakes Blvd, Mawson Lakes, Adelaide, SA 5095 Australia; 2 Dermatology Research Centre, Faculty of Medicine, The University of Queensland, St. Lucia, Australia

**Keywords:** Microsampling, Skin biopsy, Blood sampling, Minimally invasive, Point-of-care device, Microneedle

## Abstract

Conventional skin and blood sampling techniques for disease diagnosis, though effective, are often highly invasive and some even suffer from variations in analysis. With the improvements in molecular detection, the amount of starting sample quantity needed has significantly reduced in some diagnostic procedures, and this has led to an increased interest in microsampling techniques for disease biomarker detection. The miniaturization of sampling platforms driven by microsampling has the potential to shift disease diagnosis and monitoring closer to the point of care. The faster turnaround time for actionable results has improved patient care. The variations in sample quantification and analysis remain a challenge in the microsampling field. The future of microsampling looks promising. Emerging techniques are being clinically tested and monitored by regulatory bodies. This process is leading to safer and more reliable diagnostic platforms. This review discusses the advantages and disadvantages of current skin and blood microsampling techniques.

## Introduction

Microsampling is a procedure for capturing minute samples (usually <100 μL in the case of blood) from human body for analysis in a minimally invasive manner. In the diagnostic field, the improvement in molecular detection techniques in recent years has led to a smaller sample demand and is the main driver for innovation behind microsampling. This rapid development of microsampling has propelled the miniaturization of diagnostic devices, such as point-of-care (POC) devices.

While conventional sampling techniques are still necessary in disease diagnosis, they are not always practical. For instance, the conventional skin sampling approach for diagnosing skin cancer is the punch biopsy. They however may not be readily accessible to patients who live in remote areas due to personnel/logistical requirements that may subsequently delay clinical decision-making. The simplification of skin sample collection via microsampling has the potential to improve the availability of skin testing to these areas reducing the turnaround time. There is also a desire for less invasive diagnostic procedures owing to the growing concern of the patient’s individual feelings (Cohen et al. 2014; Graber et al. 2017). Microsampling plays a key role in this evolving diagnostic field as a smaller sample size has the ability to expand the potential for a minimally invasive, convenient and cost-effective diagnosis.

Microsampling and POC devices are becoming more and more popular. According to multiple market research reports, the global POC diagnostic market is expected to grow from U$23.5bn in 2016 to U$40.5bn by 2022, with a compound annual growth rate of ~10% (Global Point Of Care Diagnostics Market will reach USD 40.50 Billion by 2022: Zion Market Research n.d.; Blood Collection Market worth 10.59 Billion USD by 2022 n.d.; Point-of-Care/Rapid Diagnostics Market Worth 38.13 Billion USD by 2022 n.d.). Such a trend also has a huge impact on the business model of diagnostic services (Cohen et al. 2014; Jones et al. 2007). In this review, existing techniques for microsampling of skin or blood are evaluated. In addition, the impact of these techniques on the diagnostic field is discussed.

## Blood sampling

### Conventional blood sampling approach and its limitations

**Fig. 1:**
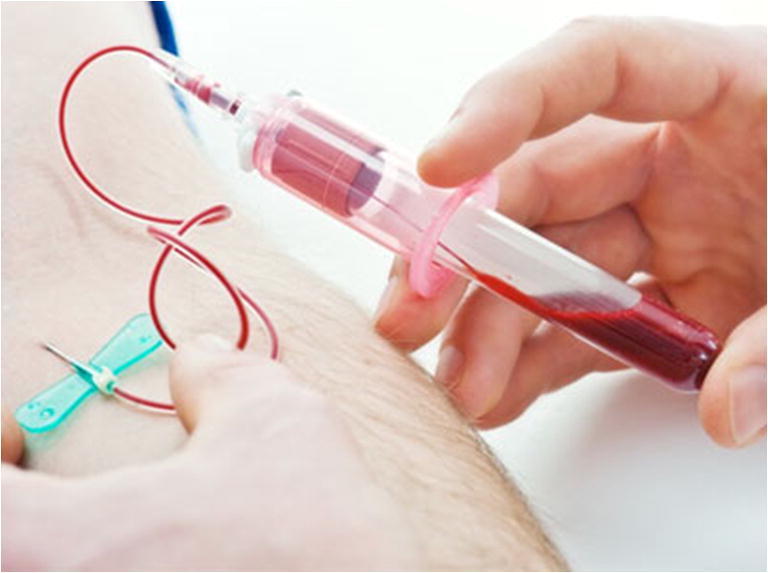
Phlebotomy is one of the most commonly used conventional blood sampling techniques. The process consists of using an invasive needle and/or catheter to sample > 0.1–1 mL of blood for biomarker analysis. Picture retrieved from https://www.butlertech.org/event/phlebotomy-program-starts/

The use of whole blood sample for biomarker detection is one of the most commonly used procedures in disease diagnosis, with an estimated global market size of U$55bn in 2017 (Global Blood Testing Market Size &amp; Share | Industry Report, 2018–2024 n.d.). The current standard for whole blood sampling necessitates a phlebotomist to collect a relatively large volume of blood (>0.1–1 mL) intravenously using a hypodermic needle connected to an evacuation tube (Freeman et al. 2018) (Fig. 1). Various types of evacuation tubes can be used based on the risk of contamination and coagulation, such as an EDTA pre-filled tube that prevents clotting (Banfi et al. 2007). Venipuncture is often performed on the volar forearm of the patient in the sampling process, and a tourniquet can be used to restrict blood flow. Once the sample is collected in the evacuation tube, it can be delivered to a central diagnostic laboratory for analysis under appropriate storage and delivery conditions (Tsai et al. n.d.).

Although the technique can provide accurate diagnosis, it is often considered a costly and time-consuming procedure due to the consumables, personnel and storage/logistical requirements (Freeman et al. 2018). For example, in a central testing laboratory setting, the turnaround time of blood testing ranges from hours to days depending on the sample/result delivery between the clinic and the central laboratory (Cohen et al. 2014; Jones et al. 2007). A central laboratory might require more than double the amount of time to process a sample as compared to a POC test for some basic blood tests, such as cell count and glucose level analysis (Tsai et al. n.d.). Another limitation of the conventional approach stems from the fact that it is tailored to be used on adults and not readily suitable for pediatric applications, such as newborn screening, as the technique is considered an invasive procedure (Freeman et al. 2018).

### Blood microsampling

With improvements in molecular analysis techniques, the demand for sample size in disease diagnosis has significantly reduced. In the case of blood, a microsample generally refers to a sample size of <100 μL as opposed to >1 mL in phlebotomy (Nys et al. 2017). A smaller sample demand addresses the limitations in conventional techniques by expanding the potential for a quicker, cost-effective and less invasive diagnostic procedure (Table 1). It is also well recognized that repeated and large-volume sampling is undesirable for patients, especially for patients with chronic disease who require regular sampling or when maintaining blood volume is critical (Chapman et al. 2014). Most microsampling approaches are less painful and stressful and often have fewer requirements on handling and storage. Therefore the benefits of blood microsampling has the potential to increase the patient’s accessibility to a blood test (Chapman et al. 2014).

#### Dried blood spot

**Fig. 2:**
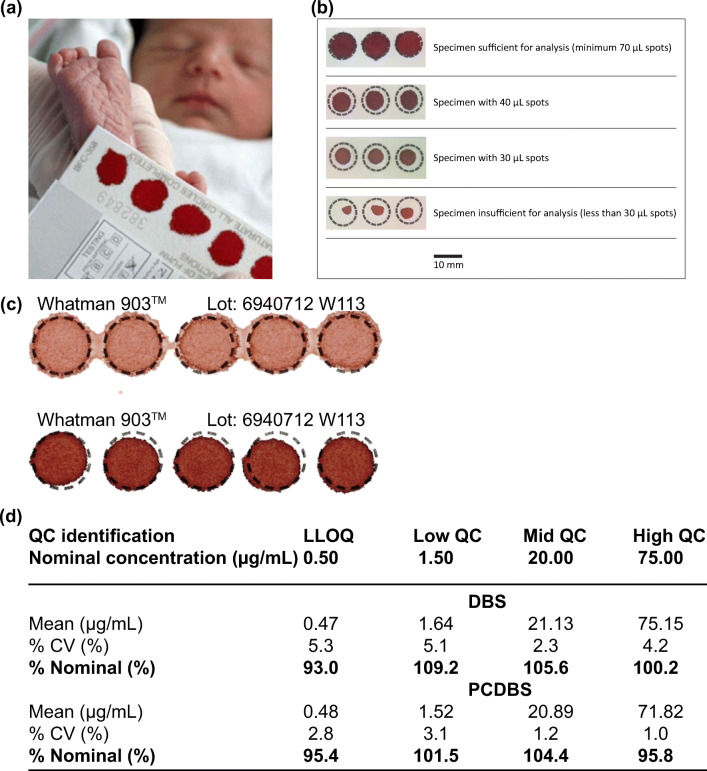
Dried blood spot (DBS) is the most common blood microsampling approach. It involves using a collection card to collect a sample of blood via finger-prick. Due to its low sample demand, it is often performed on infants (panel a). b) The volume of blood needs to be standardized in DBS for downstream analysis. Because of the strict requirement on sample volume, a technician is usually required to perform the technique. c) The hematocrit (HCT) level can lead to variations in analysis and can sometimes be distinguished by the color of sample. The HCT level of the top sample was 0.35 and the bottom was 0.5. d) Youhnovski et al. compared the precision and accuracy of DBS and PCDBS. PCDBS showed higher precision (%CV) and accuracy (%nominal). Pictures retrieved/adopted from http://www.ewbbu.com/mobile-health.html, Govender et al. 2016, Wilhelm et al. 2014 and Youhnovski et al. 2011. All figures are under a Creative Commons Attribution 2.0. Full terms at http://creativecommons.org/licenses/by/2.0

An approach that bridges the gap between small blood sampling and analysis is dried blood spot (DBS). DBS was first developed for large-scale screening of the metabolic disease, phenylketonuria, on neonates in 1963 (Guthrie and Susi 1963). Since then, it has been used for detection of sickle cell disorders and human immunodeficiency virus infection (Parker and Cubitt 1999). The technique involves the use of a blood lancet and a sample collection card. After performing a finger-prick on the patient with the lancet, a few drops of blood, usually <100 μL (Waltz 2017), can be adsorbed onto the sample collection card, followed by drying, storage and downstream diagnostic analysis (Fig. 2a).

The sample demand of DBS is much less than phlebotomy, and the experimental setup does not require any centrifugation or sub-aliquoting of plasma (Wilhelm et al. 2014). The shipping and storage requirements are simple and can be performed at ambient temperature for most downstream tests. The simple setup, flexible logistic requirements and integration with current analytical techniques have resulted in DBS being utilized routinely in large-scale epidemiological studies globally (Manakongtreecheep and Davis 2017; Easterbrook et al. 2017). DBS and heel-stick are commonly performed together on infants to obtain capillary blood due to the lower sample demand and minimally invasive nature (Patton et al. 2007).

However, the technique often suffers from variations in sample quantification and analysis due to technical errors and biological fluctuations, such as hematocrit (Hall et al. 2015) (HCT). When spotting a fixed volume of blood sample on the sample collection card, the HCT affects the resultant size of spot (Fig. 2b, c). A high HCT tends to give a smaller spot, conversely a low HCT provides a bigger spot. The HCT effect can introduce variation in analysis, such as the amount of analytes in the sample (Hall et al. 2015; Fallah and Peighambardoust 2012).

On the other hand, a pre-cut dried blood spot (PCDBS) is a variation of DBS that aims to address the HCT issue in DBS. In this technique, the entire spot of collected sample is analyzed to minimize the impact of HCT on sample analysis. This method retains almost all advantages of DBS while overcoming the impact of HCT. Youhnovski et al. reported using the PCDBS technique with a 3-mm disk punched out from DBS card for fixed volume blood absorption. Liquid chromatography/electrospray ionization tandem mass spectrometry was then performed to determine the naproxen concentration in the sample. The main objective of PCDBS was to tackle the HCT effect and drug distribution issue encountered in general DBS (Youhnovski et al. 2011). As compared to the general DBS approach, the PCDBS technique displayed lower variations in precision (%CV ≤3.1%) and higher accuracy (%nominal value: 95.4–104.4%) across the HCT range of 25% to 75%. Although the PCDBS technique partially overcame the HCT impact, the sampling process required a precise and accurate volume of blood, making it unsuitable for self-sampling without extensive training (Fig. 2d).

#### Plasma microsampling

Plasma microsampling involves collecting a small amount of plasma for quantitative analysis of biomolecules. A patient is first finger-pricked and < 100 μL of blood sample is collected into a capillary tube or a plastic device (Li et al. 2012). The sample is then centrifuged, so the plasma can be isolated and analyzed directly with techniques such as liquid chromatography - tandem mass spectrometry (Parker et al. 2015a) (LC-MS/MS). Parker et al. has reported a dried plasma spot (DPS) technique in which a small volume of harvested plasma sample was spotted onto cellulose cards for subsequent quantitative analysis of fosfomycin with LC-MS/MS (Parker et al. 2015a). The results displayed a high accuracy (<±8.5%) and high precision (<±4%) across a range of fosfomycin concentrations (Table 1).

**Table 1:** Selected publications of current blood and skin microsampling techniques

Sample target	Technology	Target model system	Application	Analytical method	Additional information	Potential for self-sampling	Sample amount	Reference
Blood	Dried blood spot	Human	Drug detection and quantification	LC/ESI-MS/MS	- Use of pre-cut filter paper to overcome hematocrit (HCT) impact - Less than ±3% of variations in volume - A precise volume of blood is necessary	No	~100 μL of whole blood	Youhnovski et al. 2011
		Human	Drug detection and quantification	LC-MS/MS	- Use of conventional filter paper card - Presence of HCT impact	No	~100 μL of whole blood	Koster et al. 2013
		Human	Virus detection	PCR	- Use of conventional filter paper card - Presence of HCT impact	No	>70 μL of whole blood	Govender et al. 2016
	Plasma microsampling	Human	Protein detection and quantification	LC-MS/MS	- Use of three-layer sample separation card - Lab setting is not necessary - Absence of HCT impact, but biological information is limited	Yes	2.5 μL of plasma from 25 μL of whole blood	Kim et al. 2013
		Human	Drug detection and quantification	LC-MS/MS	- Use of centrifugation in plasma separation - Lab setting is necessary - Absence of HCT impact, but biological information is limited	No	25 μL of plasma	Li et al. 2015a, b
		Human	Drug detection and quantification	LC-MS/MS	- Use of centrifugation in plasma separation - Lab setting is necessary - Absence of HCT impact, but biological information is limited	No	5–30 μL of plasma from 2 mL of whole blood	Parker et al. 2015a, b
	VAMS	Human	Drug detection and quantification	LC-MS/MS	- Fixed volume sampling - HCT impact can potentially be ignored - Less than ±5% of variations in volume - Sample collection within 2–4 s - Convenient sample storage	Yes	10 μL of whole blood	Denniff and Spooner 2014
		Human	Drug detection and quantification	LC-MS/MS	- Fixed volume sampling - Biased drug levels at low and high HCT levels - Sample collection within 2–4 s - Convenient sample storage	Yes	10 μL of whole blood	Mano et al. 2015
		Rat	Drug detection and quantification	LC–MS/MS	- Fixed volume sampling - Biased drug levels at low HCT level - Sample collection within 2–4 s - Convenient sample storage	Yes	10 μL of whole blood	Kita et al. 2018
	hemaPen	Human	Fixed volume sampling of whole blood	N/A	- Fixed volume sampling - HCT impact can potentially be ignored - Absence of clinical data - Overall collection duration less than 20 s - Convenient sample storage	Yes	N/A	https://www.trajanscimed.com/pages/hemapen
Skin/Blood	Microneedle	Mouse	Biomolecule detection and quantification	ELISA	- Use of solid microneedle arrays - For disposable use only - Variations in sample capturing	No	N/A	Bhargav et al. 2012
		Mouse	Drug level monitoring	Electrochemistry	- Use of hollow microneedle arrays - For continuous monitoring - Integrated diagnostic platform	No	N/A	Mohan et al. 2017
		Mouse	Biomolecule level quantification	Colorimetric assay	- Use of single hollow microneedle - For disposable use only - Integrated diagnostic platform	Yes	30 μL of whole blood	Li et al. 2015a, b
		Human	Skin sampling	qPCR	- Use of single hollow microneedle - For disposable use only - Low cost and simple design	Yes	3–12 μg of skin sample	Lin et al. 2013
		Mouse	Body fluid sampling	N/A	- Use of absorbent microneedle arrays - For disposable use only - Low cost and simple design - Use of external pressure to improve yield	Yes	~3 μL of body fluid	Samant and Prausnitz 2018
		Human	Virus detection	PCR	- Use of hollow microneedle arrays - For disposable use only - Low cost and simple design - Spatial and repeated sampling capabilities	Yes	3–12 μg of skin sample	Tom et al. 2016
		Human	Parasite detection	PCR	- Use of single absorbent microneedle - For disposable use only - Low cost and simple design - Capability to sample both skin and blood	Yes	1.5–3 μL of blood; unclear for skin.	Kirstein et al. 2017
Skin	Abrasion	Human	Skin disease diagnosis	Bacterial culture	- Use of various culturing conditions to distinguish bacteria	No	N/A	Pachtman et al. 1954
		Human	Skin disease diagnosis	PCR	- Inconsistent genotypic results between physician- and self-collected sampled	No	N/A	Hernandez et al. 2006
	Tape stripping	Human	Protein level quantification	BCA	- Great inter-individual variances in protein content	No	11 μg of protein/cm^2^	Clausen et al. 2016
		Human	Melanoma diagnosis	Microarray	- Use of 17-gene classifier to distinguish melanoma - 100% sensitivity and 88% specificity (melanomas vs naevi)	No	N/A	Wachsman et al. 2011
		Human	Melanoma diagnosis	qPCR	- Use of only 2 genes to distinguish melanoma - 91% sensitivity and 69% specificity (melanomas vs naevi) - Short turnaround time	No	N/A	Gerami et al. 2017
	Fractional skin harvesting	Swine	Skin grafting	Not applicable	- Use of modified hypodermal needle - Potential for other applications, such as tissue sampling for histopathological analysis	No	10–100 mg/skin column	Tam et al. 2013
Other bodily fluids	Reverse iontophoresis	Human	Glucose level monitoring	Electrochemistry	- Inconsistent extraction rate - Unsuitable for real-time monitoring due to reading delay - Low accuracy when the analyte concentration is low	Yes	20–60 nmol/cm^2^*h	Diabetes Research in Children Network (DIRECNET) Study Group 2003
		Human	Glucose level monitoring	Electrochemistry	- Lower variability as compared to GlucoWatch - Pixel density needs to align with follicle density	Yes	N/A	Lipani et al. 2018
	Nanopore	Human	Glucose and asparagine level monitoring	Electric signal	- Highly sensitive - Application can be limited	Yes	Glucose: 2 μL for sweat 15 μL for saliva 10 nL for blood	Galenkamp et al. 2018
	Oral rinse	Human	HPV diagnosis	PCR	- Saliva sampling - Use of 1 biomarker for HPV screening	No	N/A	Qureishi et al. 2018
	Passive drooling	Human	Subclinical malaria diagnosis	Immunoassay	- Saliva sampling - High sensitivity and specificity - Low cost	No	2 mL	Tao et al. 2019
	Wearable sweat biosensor	Human	Ethanol level monitoring	Electrochemistry	- Continuous sweat sampling - Low cost and simple design - Suitable for low-resource areas	Yes	100 nL/cm^2^	Hauke et al. 2018
	AdenoPlus	Human	Adenoviral conjunctivitis diagnosis	Immunoassay	- Disease-specific tear sampling - Shot turnaround time	No	N/A	Sambursky et al. 2013

A major advantage of plasma microsampling is sample homogeneity and absence of blood cells. Therefore, the HCT effect can be ignored in analysis (Parker et al. 2015a; Li et al. 2015a). In some cases, the concentration of molecules in plasma alone provides sufficient information for disease diagnosis. Thus, plasma microsampling is popular in some contexts as it is simple and more cost-effective than whole blood collection (Li et al. 2012).

**Fig. 3:**
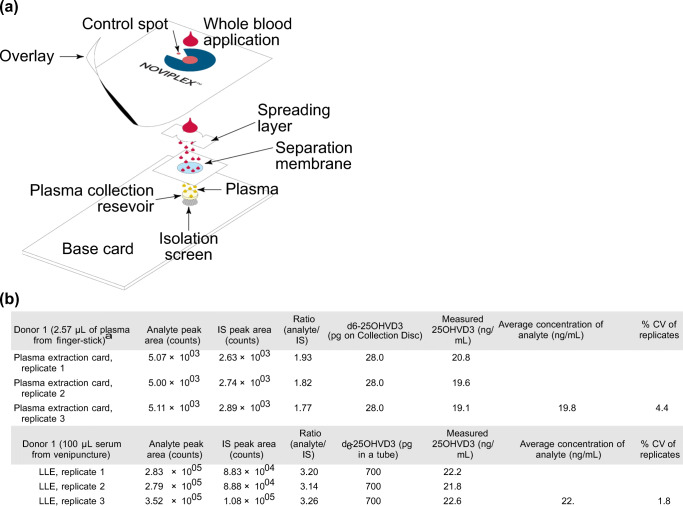
Plasma microsampling involves sampling whole blood and separating plasma out for analysis. a) The plasma collection card was designed to separate plasma from whole blood without centrifugation. b) The coefficients of variation (%CV) were similar for both the plasma extraction card and conventional liquid-liquid extraction. Reprinted (adapted) with permission from Kim et al. 2013. Copyright 2018 American Chemical Society

However, both DPS methods mentioned above require centrifugation and are only feasible in a laboratory setting. Two later studies reported plasma sampling approaches that were similar to Parker’s, but both studies utilized a multi-layer design to filter out plasma from a finger-prick blood sample without centrifugation (Li et al. 2012; Kim et al. 2013). In one of the studies, Kim et al. reported a plasma filtration card consisting of three layers; spreading layer, filtration layer and collection reservoir (Fig. 3a). The three layers were stacked together to filter out red blood cells for plasma collection (Kim et al. 2013). After loading the whole blood sample onto the spreading layer and waiting a few minutes, the spreading and filtration layers were removed. The plasma sample was formed in the reservoir at the bottom without any centrifugation.

In this study, the plasma extraction card was used to quantify the amount of Vitamin D in whole blood sample with LC-MS/MS, and the result was compared with the conventional plasma separation approach of liquid-liquid extraction across an HCT range of 21% to 70%. The result suggested that the coefficients of variation were 4.4 for the plasma extraction card and 1.8% for liquid-liquid extraction (Fig. 3b). The measured values of Vitamin D concentration were also similar (19.8 ng/mL vs 22.2 ng/mL). This showed that the results of the three-layer plasma collection card and centrifugation were comparable.

Plasma sampling via the plasma extraction card method has the potential to change the way diagnostic services are performed in resource-poor areas as a result of the reduced need for specialized laboratory equipment. Although this technique can also overcome the HCT effect on the whole blood sample, potential widespread adoption for applications could be restricted due to the limited biological information that the plasma sample may provide.

#### Volumetric absorptive microsampling

**Fig. 4:**
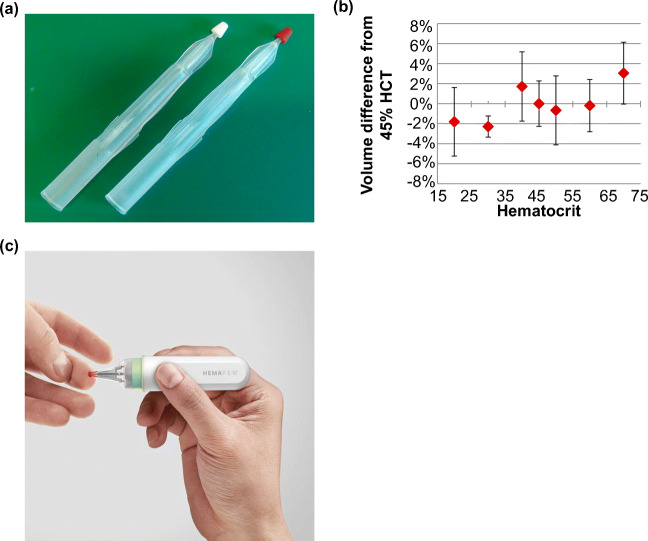
Blood microsampling with volumetric absorptive microsampling (VAMS) and hemaPEN are emerging techniques for facilitating convenient and accurate sampling. a) VAMS sticks before (left) and after (right) sampling. b) Blood sample recovered from VAMS tip displayed less than 5% volumetric variation when compared to pipetting across the HCT range of 20 to 70%. c) The application of hemaPEN following finger-prick. Pictures retrieved from Denniff and Spooner 2014 and https://www.trajanscimed.com/pages/hemapen

Volumetric absorptive microsampling (VAMS) is a novel approach that permits the collection of a fixed volume of whole blood. The technique involves the use of a sampler with a porous hydrophilic tip that enables the collection of small, accurate and precise blood volumes (~10 μL, Fig. 4a). The collection process usually takes around 2–4 s regardless of the HCT level (Denniff and Spooner 2014). After drying, the samples can be stored, transported or directly analyzed with LC/MS or immunoassay. The technique is gaining more and more attention in pharmacokinetic and therapeutic drug monitoring studies because of its simplicity and cost effectiveness (Kok and Fillet 2018). The purpose of the technique is to improve test reliability by providing fixed volume sample of blood and facilitate self-sampling with minimal instructions.

Denniff et al. has assessed the variability of VAMS in blood absorption using radioactive caffeine as a tracer *in vitro*. In the study, the blood sample was spiked with (Parker and Cubitt 1999)C-labeled caffeine for determining the amount of blood absorbed by VAMS. The radioactivity of a VAMS tip dipped in a pool of blood was then compared with another tip that absorbed 10 μL sample from pipetting. The result suggested that the VAMS tip absorbed the blood sample with less than 5% volumetric variation across a HCT range of 20 to 70% (Denniff and Spooner 2010) (Fig. 4b), as compared to 30% of variation from a DBS using the sub-punch method (Denniff and Spooner 2010).

A large number of studies suggested that VAMS can sample an accurate blood volume independent of HCT level, however, it has also been reported that the analyte recovery varies as the HCT level changes. For example, in two separate pharmacokinetic studies, Parker et al. and Mano et al. both suggested that the drug recovery levels of analytes, fosfomycin and tacrolimus, were inversely correlated with HCT levels (Parker et al. 2015b; Mano et al. 2015). Both studies examined the analyte recovery at three different HCT levels across the range of 20 to 65%. The recoveries of analytes were shown to be higher at a lower HCT level (20–30%) for both analytes. However, the main reason that led to this inverse relationship is still unclear. These studies suggested that challenges remain for analyzing drug concentration with VAMS. Although the technique provides a convenient way to sample an accurate volume of blood, the relationship between HCT and analyte needs to be thoroughly investigated before it can be used as a routine sampling tool.

Similar to VAMS, the hemaPEN (Trajan Medical and Scientific) is a sampling platform that aims to tackle volume inaccuracy and HCT effect in DBS (hemaPEN - Trajan Scientific and Medical n.d.) (Fig. 4c). The device contains end-to-end capillaries with pre-punched DBS paper for blood collection and dispensing. The purpose of the device is to provide an easy and convenient sampling experience. Upon performing a finger-prick, the device uses a capillary collection mechanism to dispense an accurate volume of blood onto filter paper. The sample integrity is ensured at every stage of the process via the integrated compartments in the device.

Following sampling, the device can be sent to a diagnostic laboratory for analysis. Since the hemaPEN is still under development, the next key step for a device like this is to demonstrate the reliability with clinical data. While the feasibility and reliability of both VAMS and hemaPEN still require further validation, the importance of accurate sampling volume and reducing the HCT impact are two of many issues both devises are aiming to address.

#### Microneedle

Unlike other established techniques, microneedles are capable of penetrating the skin and sampling at the same time without the use of a blood lancet. Early microneedle designs usually possessed a solid body and were primarily designed for cosmetic or therapeutic purposes, such as drug delivery Kim et al. 2012). Recent advances in rapid prototyping techniques, for example 3D printing and laser cutting, has enabled microsampling with microneedles for clinical research purposes (Lin et al. 2013; Samant and Prausnitz 2018).

The dimensions and geometries of microneedles can vary and can have an impact on needle penetration and pain. Reported microneedle penetration can range from 1 to 2 mm (Iriarte et al. 2017; Römgens et al. 2014). The range was sometimes comparable to blood lancet penetration depths, which usually range between 0.85 mm on newborns to 2.2 mm on adults (Fruhstorfer et al. 1999; Kocher et al. 2009). The penetration depth allows the microneedle to bypass the stratum corneum and sample the biomarkers/biological fluids directly.

**Fig. 5:**
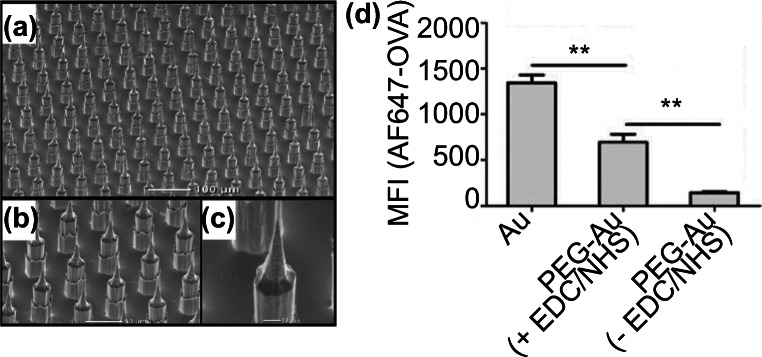
Microprojection arrays, a solid microneedle-based device, was used to capture biomarkers in the skin of live mice as reported by Bhargav et al. **a**-**c**) The surface of microprojection arrays imaged with SEM. d) Surface modifications with EDC/NHS improved the capturing efficiency. The fluorescence intensities of EDC/NHS-treated MPAs showed an 18-fold increase. Pictures retrieved from Bhargav et al. 2012

There are three major types of microneedle; solid microneedle, hollow microneedle and absorbent microneedle. Most solid microneedle designs were primarily created for drug delivery (Jenkins et al. 2012; Crichton et al. 2010), however, they can also create micropores on the skin and extract fluid/biomarkers through the resulting pores. Bhargav et al. reported a silicon chip containing an array of cone-like projections called microprojection arrays (MPAs) for selective biomarker capturing (Bhargav et al. 2012) (Fig. 5a-c). Earlier studies have already demonstrated that the MPA device was able to penetrate the dermal tissue and capture specific circulating biomarkers when applied to the skin of live mice (Crichton et al. 2010, 2011).

In the study, the impact of protein immobilization conditions on biomarker capturing efficiency was investigated, and two groups of MPAs were designed for this purpose: one group involved the use of 1-Ethyl-3-(3-dimethylaminopropyl) carbodiimide (EDC) and N-hydroxysuccinimide (NHS) in the immobilization procedure of ovalbumin on MPAs, and the other group did not use EDC/NHS. The purpose of the EDC/NHS treatment was to improve probe surface density and orientation. The resulting ovalbumin-immobilized MPA device was applied on ovalbumin-vaccinated mice to detect α-ovalbumin-IgG antibodies 21 days after vaccination. Upon application, an enzyme-linked immunosorbent assay was performed to determine the capturing efficiency of the antibody. The result showed that the EDC/NHS treatment improved the capturing efficiency of ovalbumin by 18-fold *in vivo* and 140-fold *in vitro* (Fig. 5d). However, most solid microneedle designs, including MPAs, suffered from variations in capturing efficiency and were not an ideal technology for sample quantification purposes (Ventrelli et al. 2015).

**Fig. 6:**
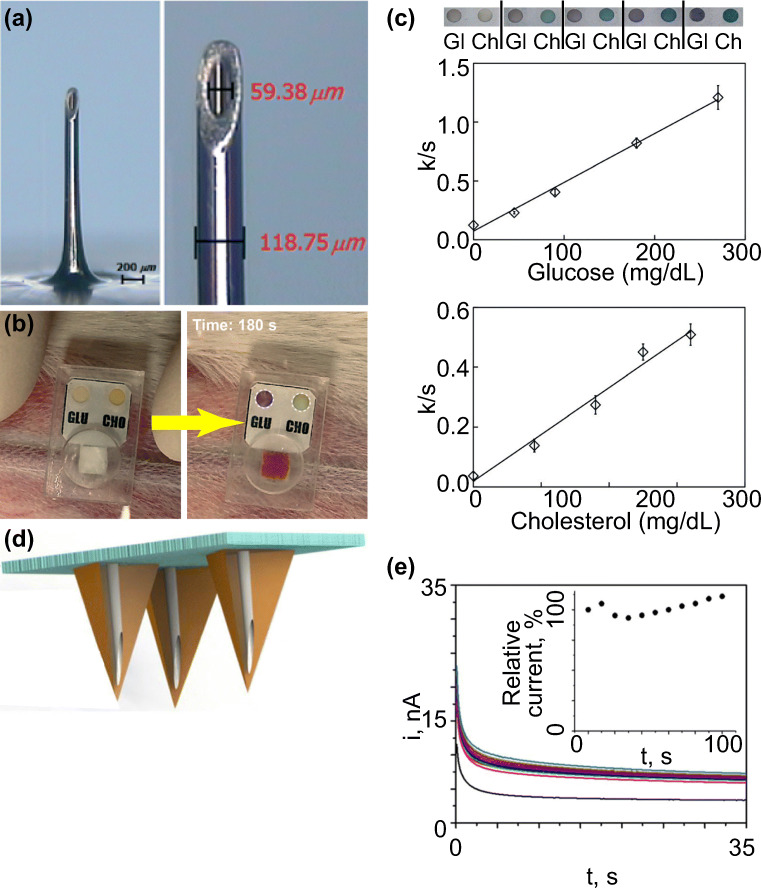
Hollow microneedles were combined with sensors for real-time biomarker monitoring. **a**) The dimension of hollow microneedle reported in Li et al. **b**) The absorption of blood led to color change in the reaction zones. The color change was used to determine the concentrations of glucose and cholesterol. **c**) The calibration curves for glucose and cholesterol measured by using the one-touch hollow microneedle device. Both measurements showed linear correlation coefficients (0.99 and 0.98). **d**) Schematic representation of the hollow microneedle array from Mohan et al. e) Real-time alcohol detection of 30 mM alcohol in artificial interstitial fluid (20.1 mg/mL BSA) for 100 min. The artificial interstitial fluid was located in a reservoir under a piece of excised mouse skin and was sampled by the microneedle array after penetrating the skin. Pictures retrieved from Li et al. 2015a, b and Mohan et al. 2017

Hollow microneedles have a channel/hollow structure for blood extraction (Kim et al. 2012). One benefit of the hollow microneedle is the potential for real-time or automated biomarker monitoring in a minimally invasive manner when paired with a wearable device. There are some wearable devices that have integrated diagnostic functionality for improved patient experience (Norman et al. 2014; Li et al. 2015b). In a recent study from Li et al., a single stainless-steel hollow microneedle with an outer diameter of 120 μm was combined with a paper-based sensor for glucose and cholesterol concentration measurement (Li et al. 2015b) (Fig. 6a and b).

Once the device was fixed to the skin of a live rabbit, the device could be activated by applying pressure at the back of the microneedle. About 30 ± 5 μL of blood sample could be collected by the hollow microneedle on a rabbit model in 3 min. The separation membrane, an asymmetric polysulfone layer, then transferred the sample through absorption to the reaction zones, which contained colorimetric assays. Color change of the reaction zones indicated the concentration of sample, and the color change was quantified by image analysis (Fig. 6b). Upon comparing different sample concentrations, the result of intensity analysis showed linear correlation coefficients (R^2^) of 0.99 for glucose and 0.98 for cholesterol, suggesting a reliable detection of sample concentration (Fig. 6c). Another advantage of the device was the use of low-cost materials, such as PDMS, which made the device ideal for disposable use. However, the technique is still at an early stage and it has only been tested on the skin of a rabbit model.

Instead of one single microneedle, a growing number of studies has reported using hollow microneedle arrays for real-time monitoring of biomarkers in blood. In most cases, a patch filled with microneedles was applied to the skin, and blood samples were continuously extracted through microfluidic channels to the back of the patch for analysis. Mohan et al. reported a hollow microneedle array coated with alcohol oxidase in the microcavities (channels) for alcohol concentration detection (Mohan et al. 2017) (Fig. 6d). Upon enzymatic reaction with alcohol, platinum and silver wires within the microcavities of needles enabled electrochemical signal measurement. The device demonstrated the ability to monitor an alcohol level of 0–80 mM for 100 min with a relative standard deviation of <2% on the skin of a mouse model (Fig. 6e). Nevertheless, the device has only been tested on animals.

Most hollow microneedle designs suffered from some common technical issues, such as channel blockage and needle deformation (Kim et al. 2012; Reed et al. 1998). Given these technical challenges, the majority of the current designs have only been performed on animals instead of humans. Another major limitation is that these designs often require the use of some advanced fabrication technologies and therefore are not scalable in most cases (Kim et al. 2018; Lutton et al. 2015).

**Fig. 7:**
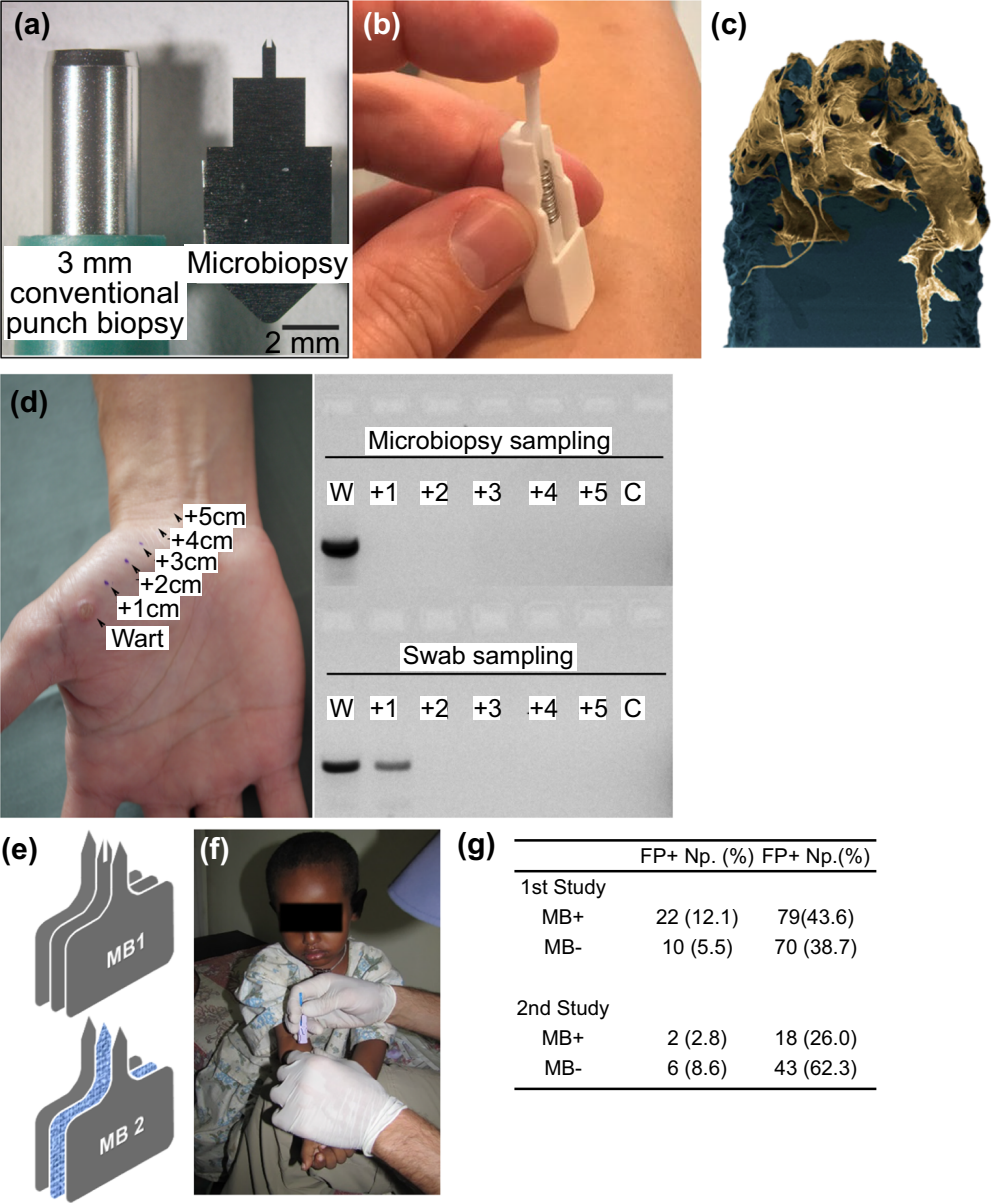
Microneedle-based microbiopsies were used in skin and blood microsampling. **a**) A side-by-side comparison between conventional punch biopsy and the skin microbiopsy. **b**) The spring-loaded applicator used in microbiopsy application. **c**) After application, the microneedle captured pieces of skin tissue in the channel. **d**) Spatial detection of HPV by sampling cutaneous warts. Skin microbiopsy provided a more accurate spatial detection as demonstrated by DNA gel electrophoresis. **e**) Designs of skin (top) and absorbent microbiopsies (bottom). The main difference between the two designs was the middle absorbent layer. **f**) The absorbent microbiopsy was used to sample patients with Leishmaniasis in rural areas. **g**) The PCR data suggested the absorbent microbiopsy was able to detect Leishmaniasis more accurately than the finger-prick method. Pictures retrieved from Lin et al. 2013, Tom et al. 2016 and Kirstein et al. 2017. All figures are under a Creative Commons Attribution 2.0. Full terms at http://creativecommons.org/licenses/by/2.0

In contrast, Lin et al. reported an alternative type of hollow microneedle, the skin microbiopsy, that was fabricated with relatively low-tech equipment, such as a laser cutter, for skin sampling (Lin et al. 2013). In the study, the skin microbiopsy was fabricated with three layers of medical-grade steel sheet (Fig. 7a, b). The skin microbiopsy penetrated the skin approximately 500 μm deep, and the channel in the middle of the microneedle was able to sample tiny pieces of skin tissue like a claw (Lin et al. 2013) (Fig. 7c). The puncture site created by the skin microbiopsy only spanned 0.2 ± 0.1 mm wide (Tom et al. 2016). The minimally invasive nature of the device also enabled spatial-temporal sampling and facilitated molecular profiling of skin diseases (Tom et al. 2016; Tan et al. 2015).

In a separate study in 2016, Tom et al. demonstrated the detection of spatial presence of human papillomavirus (HPV) with a skin microbiopsy by sampling cutaneous warts (Tom et al. 2016). After applying the skin microbiopsy with a spring-loaded applicator, 1–2 ng of DNA was extracted from the patient. The DNA gel electrophoresis result suggested that the skin microbiopsy provided a more accurate spatial detection of HPV DNA when compared to the conventional swab sampling approach, which displayed an HPV positive result for a sample collected from healthy skin 1 cm away from the lesion (Fig. 7d). Although the skin microbiopsy does not have the ability to draw blood like other hollow microneedles, the device has been tested on humans and has the potential to facilitate molecular detection of skin diseases in dermatological research.

In 2017, Kirstein et al. reported an absorbent microneedle-based device called an absorbent microbiopsy; a modified version of skin microbiopsy, for sampling of skin and blood mixture (Kirstein et al. 2017). Instead of simply pulling out skin tissue with the microneedle, the absorbent microbiopsy consisted of an absorbent layer, a layer of filter paper, in the middle to sample blood (Fig. 7e, f). In the study, the device was used to facilitate the diagnosis of Leishmaniasis, a parasitic skin condition. After applying on the lesion sites of patients, the absorbent microbiopsy collected about 1.5–3 μL of blood. Patients with histories of Leishmaniasis were sampled by both the absorbent microbiopsy and conventional finger-prick approach for comparison. Quantitative real-time kinetoplast DNA PCR data suggested that the absorbent microbiopsy was able to collect both blood and skin samples at the same time and showed a 3-fold increase (101 vs 32 patients) in accuracy in diagnosing Leishmaniasis, including asymptomatic patients (Fig. 7g).

**Fig. 8:**
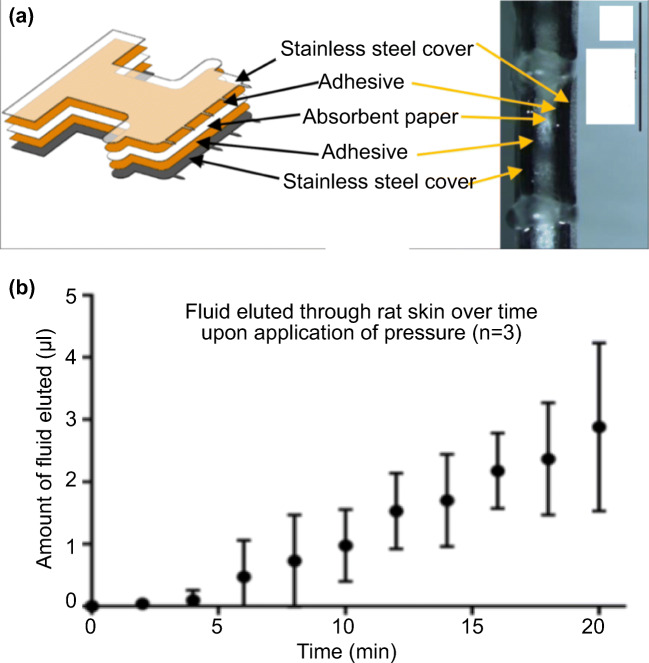
The microneedle patch reported by Samant et al. utilized absorbent microneedles to sample interstitial fluid from animal models. **a** Schematic of the microneedle patch. **b** The bottom view of the device, showing the absorbent paper was sandwiched by stainless steel covers. **c** Extraction of interstitial fluid from rat skin *in vivo*. Reproduced from Samant and Prausnitz 2018 with permission from the Royal Society of Chemistry

In addition to the absorbent microbiopsy reported by Kirstein et al., absorbent microneedles have also been reported in animal studies for dermal interstitial fluid sampling. Samant et al. reported an absorbent microneedle-based device for interstitial fluid sampling on pig and mouse skin called microneedle patch(Samant and Prausnitz 2018). The design of the microneedle patch was similar to the absorbent microbiopsy described above, with two layers of stainless-steel cover sandwiching an absorbent paper (Fig. 8a, b). However, the microneedle patch contained an array of microneedles, instead of just one single needle. After applying the patch on the skin, the patch stayed on the skin and a transverse pressure of 10 lbf was applied around the application site to increase the fluid extraction rate. At the end of the 20-min experiment, approximately 3 μL of interstitial fluid was sampled by the patch (Fig. 8c).

Absorbent microneedle-based devices such as the absorbent microbiopsy and the microneedle patch are primarily designed for single use. Although the absorbent microneedle does not offer the continuous monitoring function like the hollow microneedle, the fabrication process is usually much simpler, and thus the cost of the device is much lower comparatively. For the case of the absorbent microbiopsy, the fabrication process only involved the use of laser cutting and low-cost materials, such as filter paper and stainless-steel sheet (Lin et al. 2013; Kirstein et al. 2017). Furthermore, the absorbent microneedle has the potential to facilitate clinical research by reducing the use of reagents and the use of animals when compared to other conventional sampling approaches. Nevertheless, the sampling variation is a huge obstacle as there are a variety of factors that might affect the needle penetration and sample absorption amount, including skin thickness and microneedle dimension.

## Skin sampling

### Conventional and invasive skin sampling techniques

Another main area of microsampling is skin sampling. In skin sampling, there are a larger variety of functional techniques due to the easier access of samples. Different techniques have their own unique features, and they are often tailored for specific disease conditions. Similar to blood, most conventional approaches are invasive and expose patients to potential risk of complications.

**Fig. 9:**
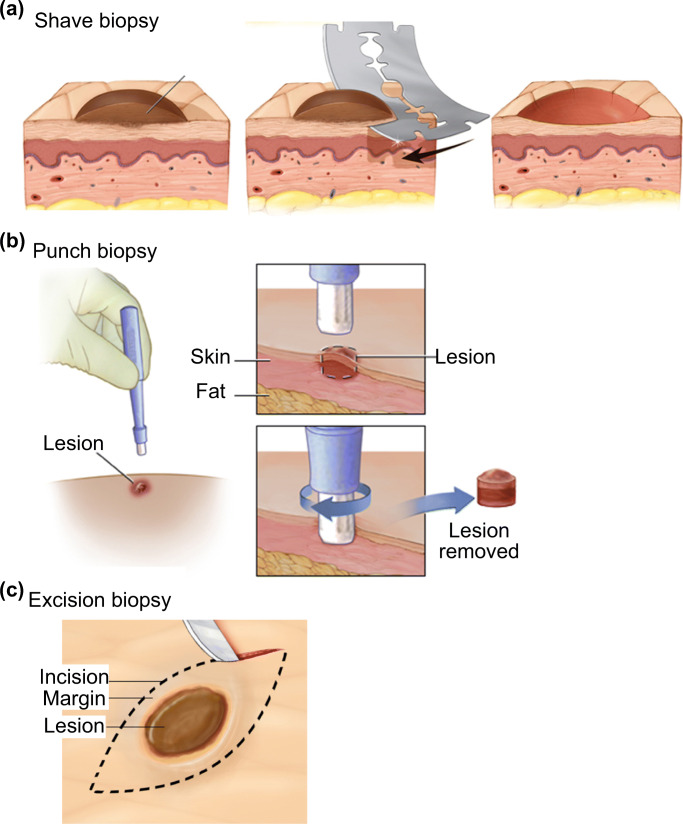
Skin biopsy is the current standard for skin conditions diagnosis. The three main types of skin biopsies are shave (**a**), punch (**b**), and excision (**c**) biopsy. All three types of skin biopsies are conducted bu a trained medical professional and the biopsy is sent for dermatopathology diagnosis in most cases. **b** The procedure for punch biopsy involves removing the suspicious location for analysis by a trained medical professional. Pictures adopted from https://www.mayoclinic.org/tests-procedures/skin-biopsy/about/pac-20384634 & https://myhealth.alberta.ca/Health/pages/conditions.aspx?hwid=hw234496

The skin biopsy is one of the most essential techniques in dermatology for diagnosis of skin conditions through histopathological assessment. In Australia alone, the cost Medicare spent on 750,000 skin biopsies in 2016 was approximately A$150 M, assuming that the minimum cost of skin biopsy and laboratory evaluation combined was A$200 per procedure (Skin cancer - Cancer Council Australia n.d.; Biopsy by a Dermatologist for moles and skin cancer n.d.). The procedure involves the removal of suspicious skin lesions followed by examination under the microscope. There are three major types of skin biopsies; punch biopsy, shave biopsy and excisional biopsy (Alguire and Mathes 1998) (Fig. 9a). All three types of biopsies are highly invasive and can cause discomfort to patients.

For instance, in punch biopsy, a doctor uses a sharp and circular tool for cutting into the top layer of fat beneath the skin (epidermis, dermis and superficial fat) and removing the suspicious site for examination. Due to the invasive nature of the technique, a suture is often needed to close the wound. The procedure needs to be performed by trained personnel, such as dermatologists. A skin punch biopsy procedure typically takes about 15 min in total. The sample may need to be sent off to a laboratory for analysis depending on the subsequent tests involved.

Although a skin punch biopsy combined with histopathological study is the gold standard for diagnosis of skin lesions, the procedure requires local anesthesia and sutures, and generally does not permit molecular analysis due to the use of formalin (Gerami et al. 2017). The procedure also requires the removal of the entire suspicious spot, irrespective of the size, which prevents any follow-up treatments or diagnosis.

Another conventional technique of skin sampling is suction blistering which involves using a chamber with a vacuum pump to extract body fluid for diagnosis of skin conditions. The extracted sample can be used to identify and quantify biomarkers. The fluid obtained with this technique is largely derived from interstitial fluid, which is the place where many important biomarkers are expected to be found. Suction blistering is much less invasive than a skin biopsy and can be used to detect drugs and several skin conditions such as epidermal necrolysis (Paquet et al. 1998), and scleroderma (Juhlin et al. 1986). However, the technique requires time (>2 h) for the blistering process and may alter the biomarker structure during the procedure.

### Skin microsampling

#### Abrasion techniques

Skin microsampling has advanced tremendously in recent years due to the lower sample demand. Some of these “new” minimally invasive techniques are in fact modifications from their predecessors, such as the abrasion technique. As one of the earliest minimally invasive skin sampling techniques, the abrasion technique was first reported by Pachtman et al. in 1954. The team utilized a wood scraper with a diameter of 23 mm to scrape the skin for sample collection from patients with Seborrheic dermatitis, an inflammatory disease, for colony counts (Pachtman et al.^1954^). The suspicious location of infection was abraded for >20 times before the sample was subsequently transferred for culturing and colony counting. The study utilized alkaline and aerobic/anaerobic environment to control the culturing conditions in order to identify different types of bacteria. Although the study was unable to find a connection between the presence of Seborrheic dermatitis and number/type of bacteria on the culture plate, the sampling methodology was adopted by the main stream specifically in clinical research settings Pachtman et al. 1954).

After Pachtman’s study, there had been a number of modified abrasion techniques for different purposes reported, including pathological studies and environmental toxin studies (Coman et al. 2014; Weldon and Du Preez 2006). However, most of these studies suffered from variations in analysis. For instance, Hernandez et al. demonstrated that samples collected via the abrasion technique by patient and physician showed inconsistent genotypic results (Hernandez et al. 2006). In the study, patients either self-sampled or were sampled by a physician for the detection of genital HPV via the abrasion technique, and the results were compared between the two groups. In the study, the collected samples were tested with PCR to detect the presence of HPV DNA. The PCR results from the two groups showed <80% of agreement for some sampling locations (Table 2).

**Table 2:** HPV detection with skin abrasion and PCR techniques as reported by Hernandez et al. The table displayed the agreement in HPV detection between paired physician-collected and self-collected male genital specimens. Glans, Shaft and scrotum all showed agreement <80%. Pictures retrieved from Hernandez et al. 2006

Site and result for physician collected samples	No. of Self-collected samples		Agreement	
	HPV^+^	HPV^-^	Overall (%)	Kappa (95%CI)
Gland/coronal sulcus				
HPV^+^	23	12	79.0	0.15 (0.33-0.68)
HPV^-^	12	67		
Shaft				
HPV^+^	43	22	67.2	0.34 (0.18-0.51)
HPV^-^	20	43		
Scrotum				
HPV^+^	31	15	69.0	0.36 (0.19-0.53)
HPV^-^	21	48		
Foreskin				
HPV^+^	6	1	95.0	0.89 (0.67-1)
HPV^-^	0	13		

This implied that variations existed between protocols and performers, and the technique should ideally be performed by trained personnel, which is one of the main reasons why the technique is not being widely used today.

**Fig. 10:**
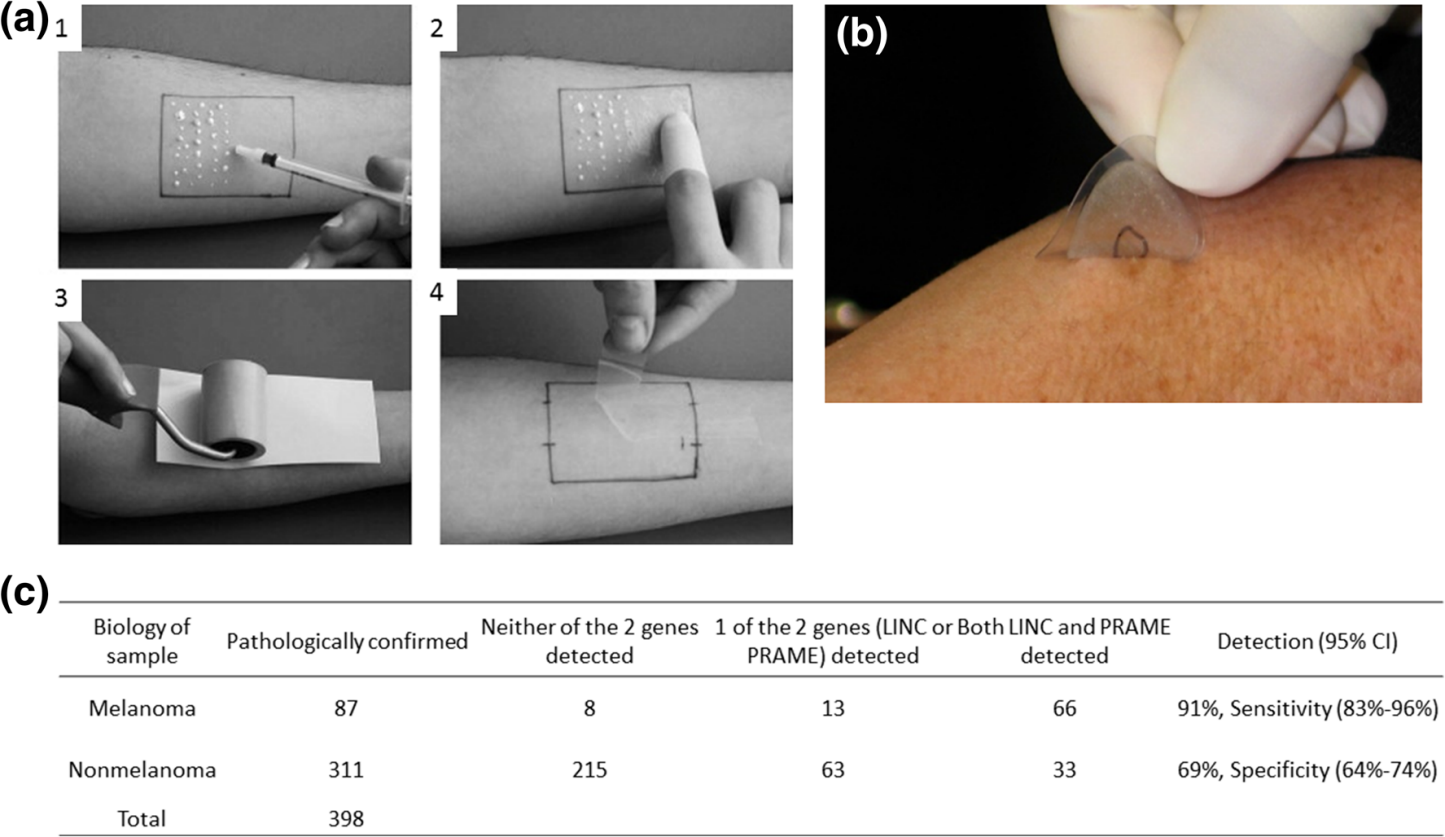
Tape stripping is a non-invasive technique for skin sampling. **a** The typical procedure of conventional tape stripping. 1) The penetration formulation was applied on the surface of the skin. 2) The formulation was spread across the sampling area. 3 & 4) The tape was applied and subsequently removed for downstream analysis. **b** The tape stripping biopsy kit from DermTech. The technique was performed by a technician at a clinic, and the tape was sent to a laboratory for analysis. **c** Performance of *LINC00518* and/or *PRAME* preferentially expressed antigen in melanoma detection in the validation sets suggested a 91% sensitivity and 69% specificity. Both numbers were higher than using a dermoscopy alone. Pictures retrieved from Lademann et al. 2009 and Gerami et al. 2017

#### Tape stripping

Skin stripping is a simple yet efficient method for stratum corneum materials sampling. It involves applying an adhesive tape on the location of interest and peeling off multiple times to sample materials on the skin surface (Fig. 10a). Conventional tape stripping usually consists of using a penetration formulation, such as an ethanolic or oily formulation, to assist sample collection. Initially, tape stripping was developed for detecting bacterial infection, with the sample directly transferred to an agar plate for culturing (Bolton et al. 1980). Recent development involved using sampled RNA and protein from stratum corneum for molecular analysis of diseases (Escobar-Chávez et al. 2008; Clausen et al. 2016). This sampling technique provides a quick and robust method of diagnosing dermatitis and drug level. However, there is no standard protocol for tape stripping and the penetration formulation varies also.

**Fig. 11:**
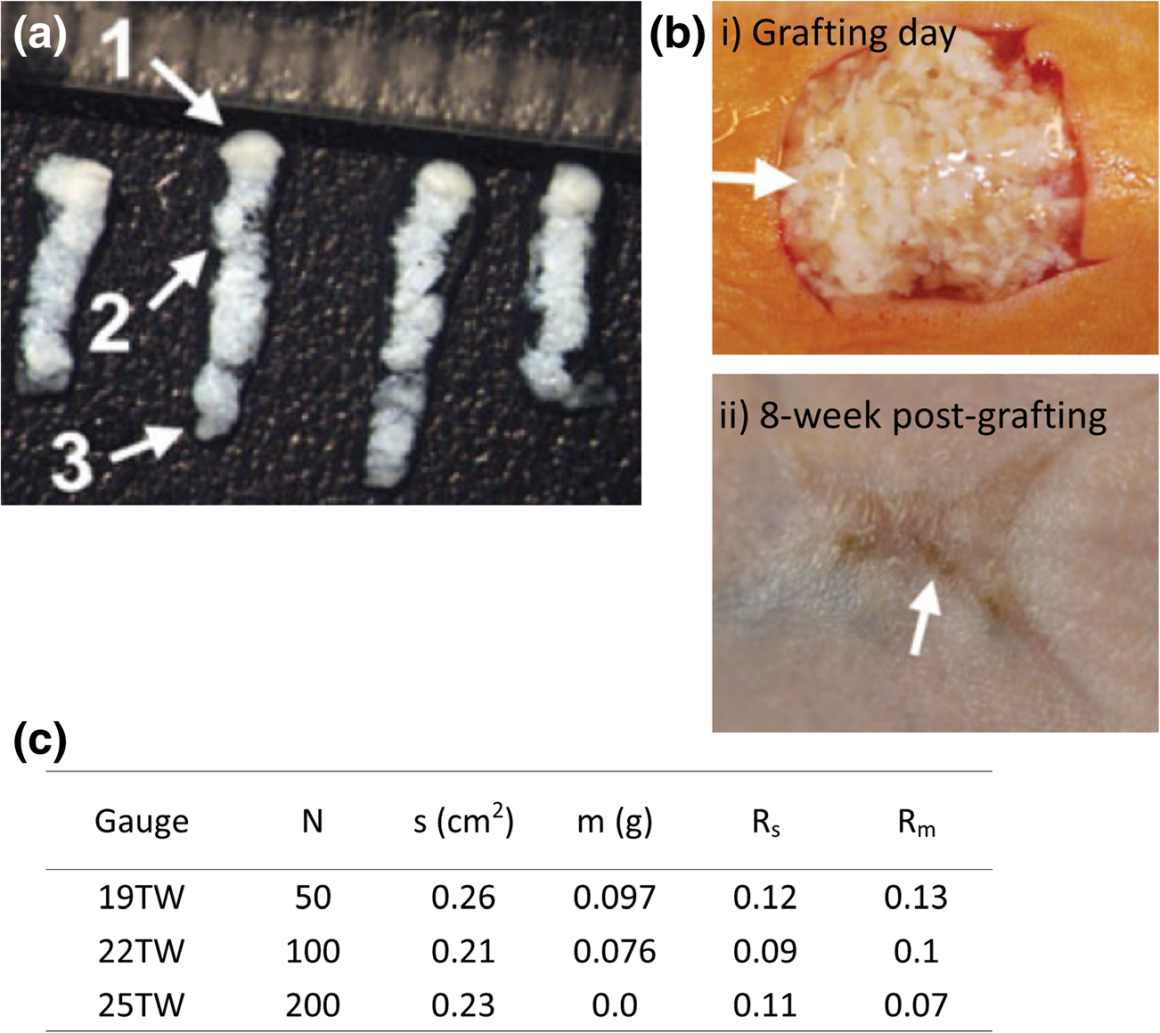
Fractional skin harvesting (FSH) was designed for skin grafting. **a** Full-thickness skin columns harvested with a 19-gauge coring needle from the donor. (Cohen et al. 2014) Epidermis; (Graber et al. 2017) dermis including adnexal structures; (Global Point Of Care Diagnostics Market will reach USD 40.50 Billion by 2022: Zion Market Research n.d.) subcutaneous fat. Each mark on the ruler in the photograph spans 1 mm. **b** The recipient site i) before and ii) after the 8-week recovery time. i) Human skin columns were applied in random orientation to a full-thickness wound on the dorsal skin of a mouse; arrow highlights the epidermal head of one skin column. ii) The recipient site formed a ‘fishing net’ in the middle (arrow) after recovery. **c** The FSH resulted in samples with smaller surface area and mass than conventional split-thickness skin graft. R_σ_ = surface area ratio; R_m_ = mass ratio. Pictures retrieved/adopted from Tam et al. 2017. All figures are under a Creative Commons Attribution 2.0. Full terms at http://creativecommons.org/licenses/by/2.0

One main advantage of tape stripping is its simplicity, the minimal setting requires little training and the potential to address a larger population. One representative example is the non-invasive skin biopsy tape from DermTech (Wachsman et al. 2011) (Fig. 10b). The technology is based on tape-stripping and is designed for the diagnosis of deadly melanoma. Conventional approaches for melanoma diagnosis include the use of 4–6 mm diameter punch biopsy or a deep shave biopsy, and the technique is highly invasive. In contrast, DermTech’s adhesive tape biopsy permits minimally invasive sampling of cells from the surface of a patient’s skin. After sampling, the tape can be stored and sent to DermTech’s laboratory for analysis. RNA extracted from the sample can be analyzed for the 17 genes that form the genetic signature for melanoma. The approximate turnaround time from sampling to result delivery to the doctor/clinic is three days (Ferris et al. 2015).

Out of the 17-gene classifiers that separate melanoma and non-melanoma, *LINC0518* (Long Intergenic Non-Protein Coding RNA 518) and *PRAME* (preferentially expressed antigen in melanoma) were one of the best performing gene pairs in identifying melanoma (Gerami et al. 2017). Gerami et al. reported using the pair of target genes combined with DermTech’s adhesive tape biopsy to distinguish melanoma from non-melanoma samples. The study was designed with 157 training sets and 398 validation sets of adhesive tape biopsy. After non-invasive sampling with the adhesive tape biopsy, the lesions were sampled with conventional biopsy for comparison with the validation sets of adhesive tape biopsy. Out of the 398 validation sets (87 melanomas and 311 non-melanomas), the qPCR result showed that *LINC00518* and/or *PRAME* detection differentiated melanoma from non-melanoma samples with a sensitivity of 91% and a specificity of 69% (Fig. 10c). Both sensitivity and specificity were higher than dermoscopy (Ferris et al. 2015).

Although DermTech’s adhesive tape biopsy needs to be performed by medical professionals, the training requirement is much lower than conventional biopsies due to its ease-of-use and non-invasive nature, making melanoma diagnostics more accessible to the public. In addition, the technique allows for repeated diagnosis and simpler disease progression monitoring. These advantages are ideal for melanoma management as melanoma in some instances can be curable if detected and treated early without going through any removal procedure(Gerami et al. 2017).

#### Fractional skin harvesting

There are also skin microsampling techniques that are designed for medical purposes other than diagnosis. A recent study from Tam et al. reported a needle-based technique called fractional skin harvesting (FSH) for skin grafting (Franco et al. 2014). FSH consists of two main parts: a standard hypodermal needle for harvesting and a fluidic device for sample collection. The harvesting needle is produced by honing a standard needle to fabricate two cutting edges. Once the needle is inserted into the skin, a column of microscopic skin tissue can be extracted. A fluidic device attached to the other end of the needle is then utilized to remove the sample from the harvesting needle with negative pressure, and the sample is transferred to a collection basket for subsequent skin grafting. The purpose of the device was to harvest and transfer hundreds of microscopic skin tissue columns directly into a skin wound.

In a later study, the device was tested on human skin for sample collection (Tam et al. 2017) (donor). The collected skin columns (Fig. 11a) were grafted on the dorsal skin of a mouse (recipient). The healing responses of both donor site and recipient site were observed over time. After grafting, the donor site healed by tissue remodeling without scarring as the wound sites were much smaller than the conventional split-thickness skin graft technique. However, the FSH technique led to inferior cosmetic outcome on the recipient as the skin wound healed resembling a ‘fishing net’ (Fig. 11b panel i & ii). The sample size of FSH was also compared with the conventional approach (Fig. 11c). Depending on the choice of needle, FSH was able to sample about 10–100 mg of skin sample with similar penetration as split-thickness skin graft while preserving the sample structure and viability. Both surface areas and masses of FSH samples were at least 8-fold smaller than split-thickness skin graft samples. This demonstrated the lower level of invasiveness of FSH. The FSH technique also has other potential applications, such as tissue sampling for histopathological analysis or tissue removal for drug delivery through the fractional channels (Franco et al. 2014; Tam et al. 2013).

**Fig. 12:**
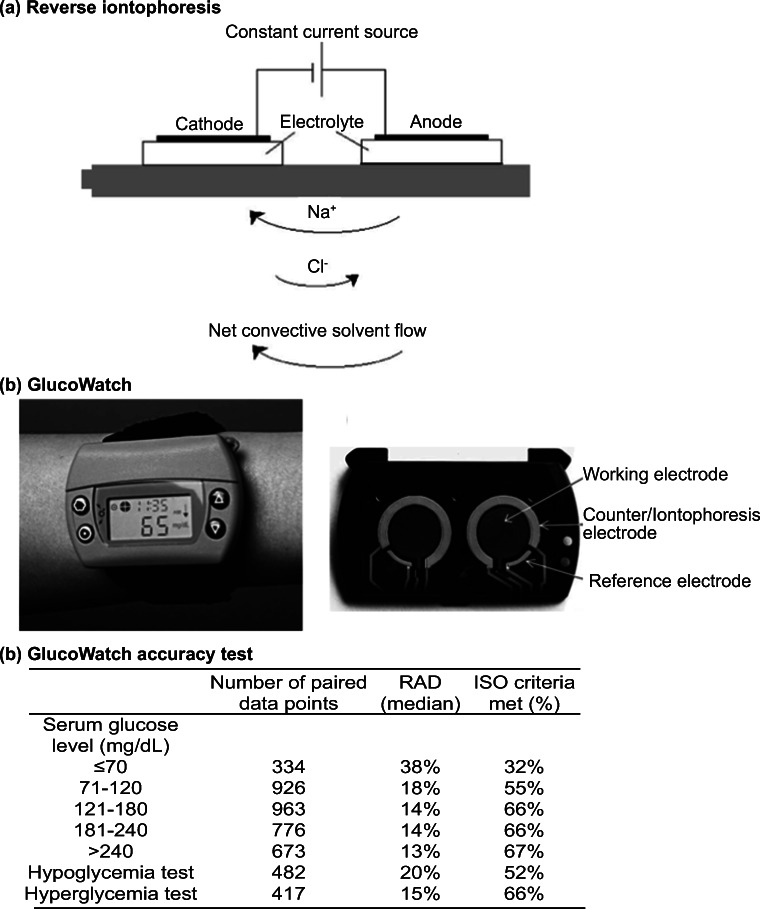
Reverse iontophoresis was utilized in glucose monitoring. **a** In reverse iontophoresis, an electrical current is applied on skin to electro-osmotically drive interstitial fluid, which usually contains targeting analytes, through the epidermis to the skin surface for analysis or sample collection. **b** The GlucoWatch was one of the reversion iontophoresis-based diagnostic platforms for glucose level monitoring. The device was separated into two pieces, a watch (panel i) and an electrode assembly (panel ii). **c** GlucoWatch model GW2B accuracy test across a range of glucose concentrations. The medium relative absolute difference (RAD) value at the lowest glucose concentration (≤70 mg/dL) was the highest among all testing concentrations. Only 32% of the data collected met the ISO criteria (±20%). Pictures retrieved/adopted from Potts et al. 2002 and the Diabetes Research in Children Network (DirecNet) Study Group

## Sampling of other bodily fluids and other technologies

### Reverse iontophoresis

Reverse iontophoresis utilizes an electric current to remove molecules and interstitial fluid through the skin for analysis (Fig. 12a). The technique involves applying a low-level electrical current, which increases the permeability of the skin. As a result, molecules are transported across the stratum corneum by electrophoresis and electro-osmosis. The transport of molecules is usually measured in units of chemical flux. The technique was first described by Glikfeld et al. for extracting glucose, theophylline and clonidine (Glikfeld et al. 1989). It was later combined with a glucose sensor in a real-time monitoring device called GlucoWatch Biographer (Ackerman et al. 1999) (Cygnus, Inc., Fig. 12b).

**Fig. 13:**
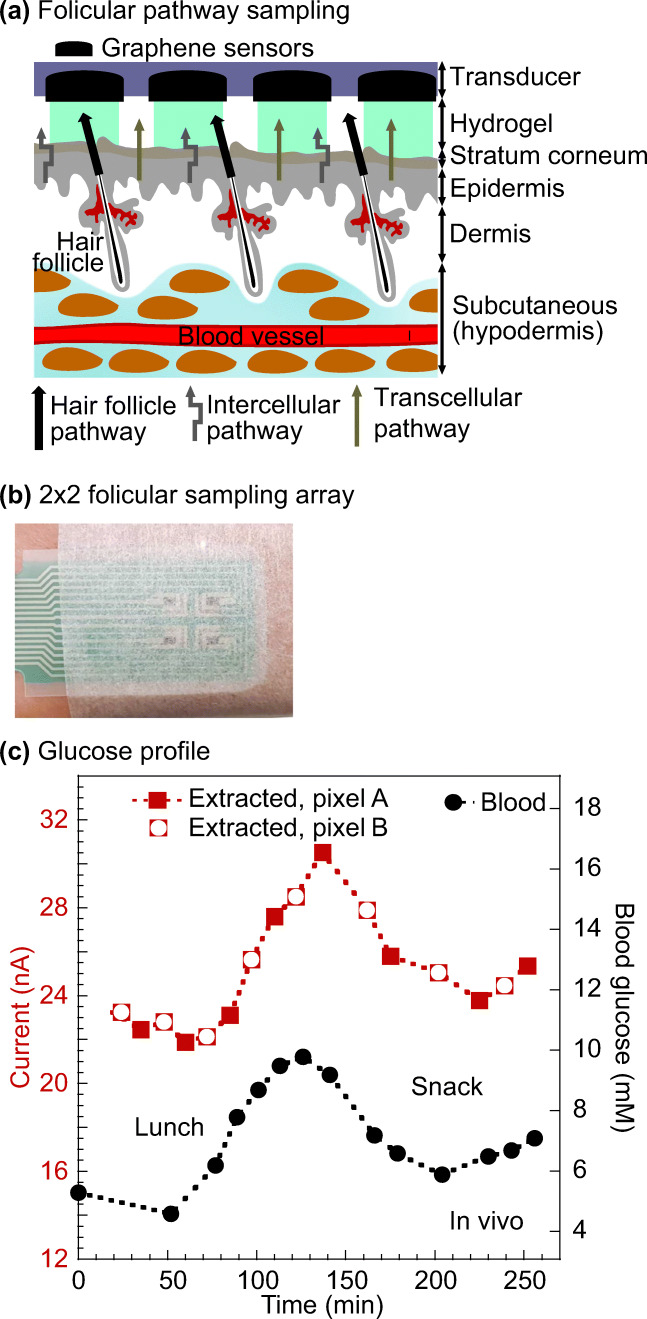
The use of follicular pathway in bodily fluid sampling provided a calibration-free glucose monitoring platform. **a** Schematic illustration of the glucose preferential pathway (hair follicles). **b** A screen-printed array fixed onto a volunteer’s forearm. The array was connected to a potentiostat. **c** The platform enabled good tracking of the blood glucose as compared to commercial platform. Pictures retrieved from Lipani et al. 2018

GlucoWatch is a wearable and non-invasive glucose monitoring device that tracks the glucose level in adults. The device requires the user to apply a plastic patch on the skin for periodic sample collection through reverse iontophoresis. Upon sample extraction, the electrochemical signal from the patch is transmitted wirelessly to a separate watch-like device that delivers the glucose level reading (Ackerman et al. 1999).

Several major problems exist with the device, including time delay and disruption by sweating (Diabetes Research in Children Network (DIRECNET) study group, T. D. R. in C. N. (DirecNet) S, 2003). In a report from Diabetes Research in Children Network (DirecNet) Study Group, the functionality and reliability of GlucoWatch were examined (Diabetes Research in Children Network (DIRECNET) study group, T. D. R. in C. N. (DirecNet) S 2003). On one hand, 60% of the sensor glucose values were within 20% of the serum glucose, suggesting an acceptable level of accuracy (data not shown). On the other hand, the measurement was less accurate at low glucose level. As shown in Fig. 12c, for a glucose level lower than 70 mg/dL, only 32% of the readings met the International Organization for Standardization (ISO) criteria (Diabetes Research in Children Network (DIRECNET) study group, T. D. R. in C. N. (DirecNet) S 2003) (±20%). This indicated that the device might only be sufficient for identifying the trends of glucose level in hyperglycemia patients unless a highly sensitive analytical method is used, which is usually difficult to achieve on a wearable device.

Another major disadvantage of GlucoWatch is the calibration procedure. A drop of finger-prick blood was required for regular calibration due to the variations caused by large sampling area (>3cm^2^ and sample dilution (Sieg et al. 2004). Lipani et al. reported an alternative reverse iontophoresis-based glucose monitoring platform without the need for regular blood calibration (Lipani et al. 2018). The device consisted of hydrogel, electrochemical glucose sensor and miniaturized electrodes (Fig. 13a). The platform exploited the fact that most of the electroosmotic flow during the reverse iontophoresis process follows low-resistance pathways associated with hair follicles. Using the graphene-based pixel arrays in the design, the interstitial fluid was extracted along the hair follicle pathway into a separate, small-volume pixel. The subsequent reaction with glucose oxidase was detected in the pixel by electrochemical sensors. By fixing the area and volumes of the pixels, sensors could operate on an individual hair follicle. The dilution factor of the extracted glucose was constant. Therefore, the inter- and intra-skin variabilities were reduced without the use of blood.

**Fig. 14:**
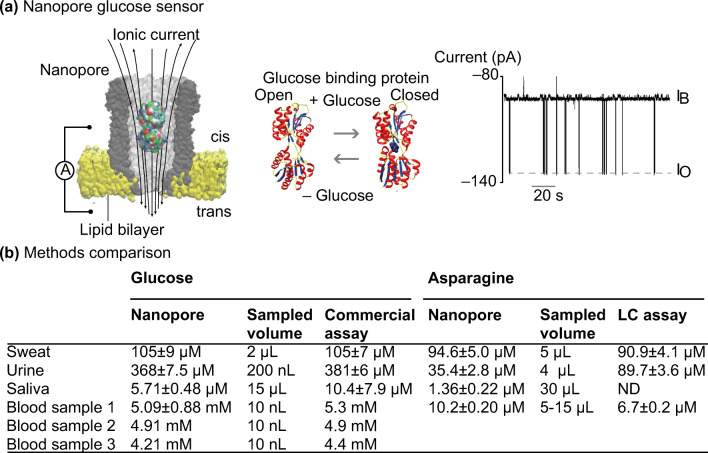
Nanopore biosensor for detecting analytes in bodily fluids. **a** Schematic illustration of the nanopore technique. The binding of analyte (glucose in this case) caused conformational change. The passing of proteins through the nanopore led to a change in current. **b** The glucose quantification with nanopore was similar to the commercial glucose meter in general. The asparagine measurement was less similar to the benchmarking technique HPLC but demonstrated a higher sensitivity in saliva sample. Pictures retrieved from Galenkamp et al. 2018. All figures are under a Creative Commons Attribution 4.0. Full terms at http://creativecommons.org/licenses/by/4.0

Figure 13b illustrated a 2 × 2 array strapped to the volunteer’s forearm. An *in vivo* experiment was performed on two healthy volunteers for comparing the glucose level tracking ability of the array with a commercial glucose meter. The experiment monitored the glucose level over the course of 6-h, which included lunch and snack periods. The interstitial fluid glucose profile collected with the array closely followed that of the blood glucose data obtained with the glucose meter (Fig. 13c). There was also an expected 15-min lagging time. This suggested the approach was able to effectively track the glucose level through interstitial fluid without any calibration.

The technique provides a more user-friendly experience when compared to GlucoWatch. One main future challenge for the platform is to ensure each pixel only interrogates one follicle. When there is more than one follicle per pixel, it may lead to the variability issue observed in GlucoWatch (Sieg et al. 2004). This can potentially be achieved by adjusting the array density. However, it is crucial to keep in mind that the follicle density varies depending on individuals (Sinclair et al. 2005). The array density may need to be adjusted accordingly.

### Nanopores

Most electronic POC biosensors rely on the oxidation of a target biomarker to generate an electric signal (Bayley 2015). However, oxidase enzymes may not be readily available for some analytes. Nanopore is an emerging technology that can address the problem. These pores are nanometer-sized water conduits spanning biological or artificial membranes (Bayley 2015). The technology also involves the use of analyte-binding protein. The binding of analyte causes a change in conformation and a current passing through the nanopores. When an electronic sensor is attached to the nanopore membrane, biological signals are converted into electrical signals for the direct quantification of analytes.

In a recent study, Galenkamp et al. reported using nanopores to detect glucose and asparagine on a real-time basis (Galenkamp et al. 2018) (Fig. 14a). The study utilized specific metabolite-binding proteins, glucose-binding protein and a substrate-binding domain that recognizes asparagine, within the nanopore cavity for binding glucose and asparagine, respectively. The glucose data was compared with the commercial glucose meter data, and the asparagine data was compared with the HPLC data.

**Fig. 15:**
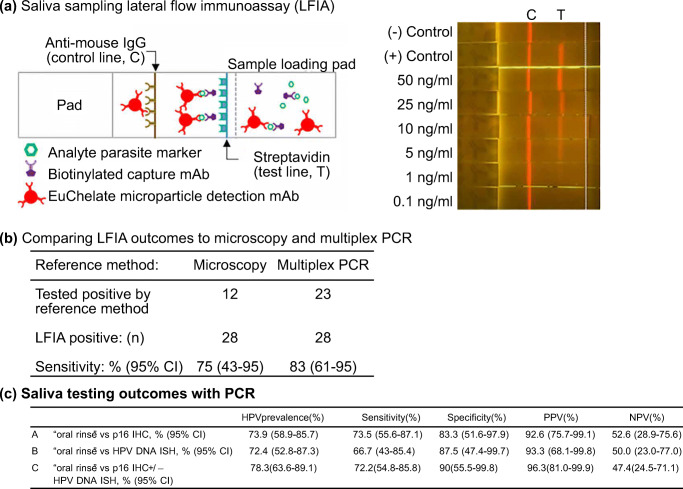
Saliva sampling for disease diagnosis. Tao et al showed that oral rinse saliva sampling followed by lateral flow immunoassays (LFIA) were able to detect malaria infection in volunteers (**a**) and (**b**). PCR was used by Qureishi et al to detect Human Papilloma virus infection using saliva testing (*c*). mAb, monoclonal antibody; CI, confidense interval; DNA ISA, DNA *in situ* hybridisation; p16 IHC, p16 immunohistochemistry; PPV, positive predictive value; NPV, negative predictive value. **b** Table estimating the sensitivity of the lateral flow immunoassay as reported in Tao et al. 2019. LFIA, lateral flow immunoassay. Pictures retrieved/adopted from Qureishi et al. 2018 and Tao et al. 2019. All figures are under a Creative Commons Attribution 4.0. Full terms at http://creativecommons.org/licenses/by/4.0

The measurement of glucose concentration with nanopores was similar to the commercial glucose meter, with less than 5% of difference across most biological samples (sweat, urine and blood samples; Fig. 14b). For the case of asparagine, the measurement with nanopores was not as close to its benchmarking technique HPLC. However, it was crucial to note that the nanopore approach was more sensitive than HPLC and was able to detect an asparagine concentration of 1.36 ± 0.22μM in saliva sample.

The nanopore technique is highly sensitive due to its single-molecule nature of the sensor. Another recent study utilized the sensitivity of nanopore and combined the technique with artificial intelligence for detecting the electrical signature of virions and facilitate influenza detection(Arima et al. 2018). One of the biggest weaknesses of this technique is the need for identifying the appropriate binding proteins for target analytes (Galenkamp et al. 2018). The analyte-binding protein also needs to work with the nanopore in order to generate any electrical signals. This can limit the technique from addressing a wider range of diagnostic areas.

### Saliva

Saliva sampling is used to aid the diagnosis of both oral and systematic diseases (Malamud and Rodriguez-Chavez 2011). The main advantages of saliva sampling include its non-invasive nature and ease-of-access to the sample. Common saliva sampling approaches include passive drooling, oral rinsing and oral swab (Avataneo et al. 2019). Initially, the development of saliva sampling diagnosis was hampered by the lack of attention to sample collection and the low amount of analytes in the sample (Malamud and Rodriguez-Chavez 2011; Avataneo et al. 2019). The improved sample handling approach combined with sensitive analytical methods, such as mass spectrometry and qPCR, has enhanced the usefulness of saliva sampling (Malamud and Rodriguez-Chavez 2011).

For oral diseases, saliva sample has been reported for diagnosing oral cancer, fungal disease and virus infection (Zheng et al. 2010; Ghannoum et al. 2010; Andrews et al. 2009). Qureishi et al. reported diagnosing HPV in patients with oropharyngeal squamous cell carcinoma (OPSCC) with the aid of saliva sample(Qureishi et al. 2018). To collect the sample, patients were required to rinse their mouth with sodium chloride solution for 20–30 s. The saliva samples from 46 patients were collected, and PCR was performed to detect the presence of HPV DNA. The result showed that the oral rinsing approach has a sensitivity and specificity of 72.2% and 90%, respectively, when compared to the reference tests immunohistochemistry and DNA *in situ* hybridization (Fig. 15a). The result was promising and suggested that a positive test could avoid the conventional and more invasive biopsy procedure.

**Fig. 16:**
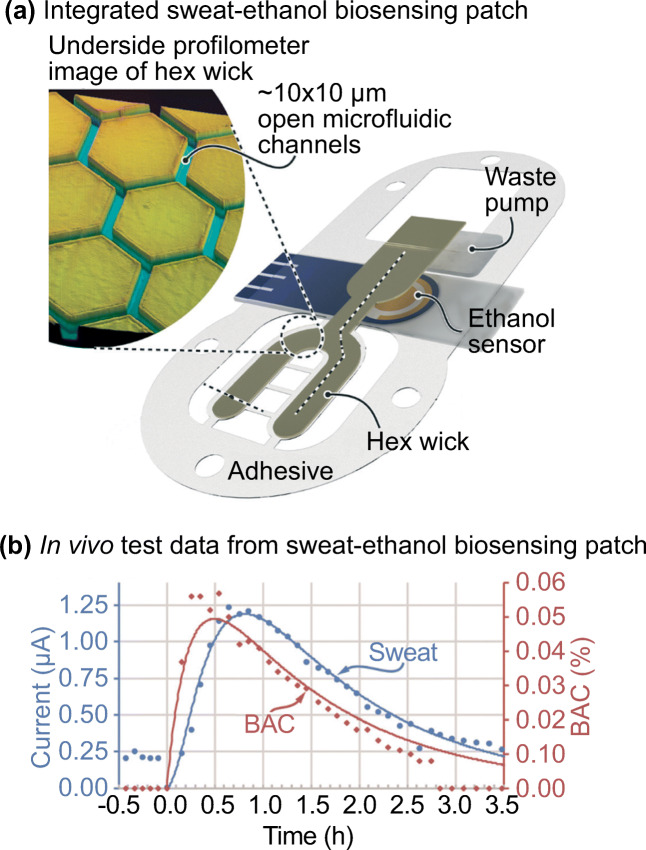
Sweat sampling with a wearable device. **a** Schematic illustration of the wearable device as reported by Hauke et al. 2018. b) *In vivo* test data and pharmacokinetic model curves for one of the testing subjects. Pictures adopted from Hauke et al. 2018. All figures are under a Creative Commons Attribution 4.0. Full terms at http://creativecommons.org/licenses/by/4.0

For systematic diseases, saliva sampling has been adopted in drug monitoring studies and epidemiological studies (Avataneo et al. 2019; Tao et al. 2019). Tao et al. reported an approach for the rapid detection of subclinical malaria infection based on the patient’s saliva sample (Tao et al. 2019). A proteomic analysis of saliva samples was first performed for the identification of a single malaria protein marker found on subclinical individuals. Then, a lateral flow immunoassay-based rapid diagnostic test was developed to detect the signature protein marker in the saliva sample collected with passive drooling. Figure 15b showed that the sensitivities of platform were 75% (microscopy as the reference) and 83% (multiplex PCR as the reference). This suggested the saliva sample combined with the lateral flow immunoassay was able to identify subclinical malaria reservoirs. The use of saliva sample instead of blood for the screening of reservoir carriers could hugely facilitate the diagnosis and prevention of malaria diagnosis in resource-poor areas.

### Sweat

Sweat has recently been adopted as a candidate for drug monitoring(Avataneo et al. 2019). Conventional approaches usually involve sampling with a sampler (Avataneo et al. 2019; Bariya et al. 2018). The simplest version of a sampler is a patch consisting of 2–3 layers of filter paper (Kintz and Samyn 2000). Due to the large size of the patch, the sampling procedure is often time-consuming and ineffective. Oftentimes, these patch samplers suffer from evaporation (Brunet et al. 2008). The repetitive swiping step may even cause irritation to the patient’s skin. To avoid these issues, Appenzeller et al. demonstrated the use of an air- and water-tight sweat collection bag for retaining the sweat sample (Brunet et al. 2008). However, the setup and recovery steps were not straightforward.

There are more and more emerging wearable devices that can analyze a patient’s sweat sample continuously (Mohan et al. 2017; Bariya et al. 2018; Kim et al. 2018; Li et al. 2019). Hauke et al. reported a wearable sweat biosensing device for monitoring ethanol concentration (Hauke et al. 2018). The device stimulated sweat by iontophoresis and absorbed the sweat sample with a patterned polyethylene terephthalate film called ‘hex wick’. The hexagonal pattern of the hex wick minimized the sample use to ~100 nL/cm^2^ (Fig. 16a). The hex wick then transferred the sample directly to the electrochemical sensor. The device continuously measured sweat concentration at 25 s intervals. The data collected was compared with blood ethanol level over a period of 3 h. The result indicated that there was a high linear Pearson correlation of 0.9474–0.9996 between the device and known pharmacokinetic models with a blood-sweat lag time ~6.5 min on average (Fig. 16b). The novelty of the design mainly came from the sample absorption ability of the hex wick. The ability to handle a small sample volume without significant sample contamination addressed one of the main issues in the field (Avataneo et al. 2019).

**Fig. 17:**
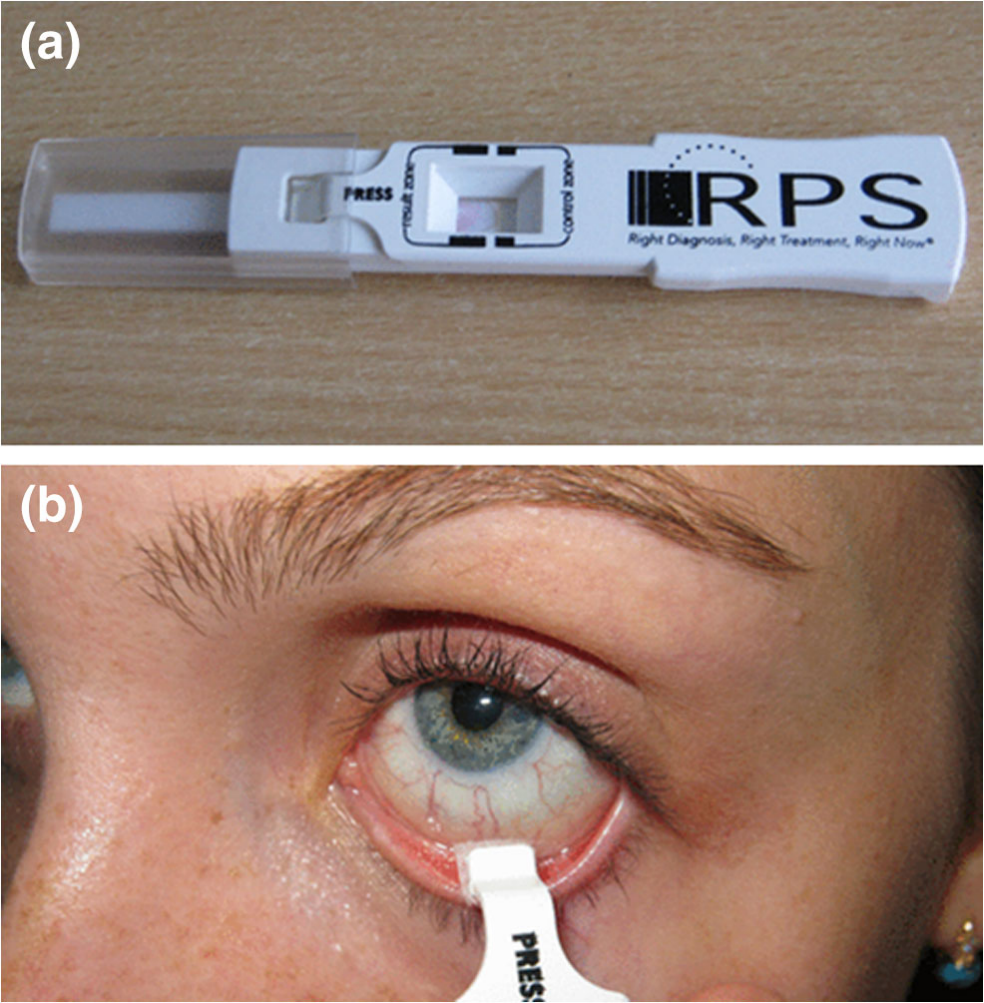
AdenoPlus test for adenoviral conjunctivitis diagnosis. **a** AdenoPlus test includes both sampling and diagnosis. **b** The sample collector with the sampler. Pictures retrieved from Kam et al. 2015

### Tear

Tear sampling is the gold standard for diagnosing the ophthalmic disease or monitoring the use of topical ophthalmic drugs (Avataneo et al. 2019; Posa et al. 2013). Comparing to saliva and sweat, tear sampling is less prone to contamination. However, the sample volume can be difficult to determine unless a stimulation approach is adopted to increase the volume (Capiau et al. 2016). The unstimulated approach hugely relies on the collection of basal tears. The conventional sampling approach for basal tears usually requires a technician to hold a capillary in the conjunctival sac and to sample with a swab or filter paper straight from patient’s eye. The approach can be unpleasant for some people. The technician may even need to collect the sample from the cheeks of non-cooperative patients (Capiau et al. 2016). However, the volume of basal tears is often insufficient for diagnostic purposes. Alternatively, a larger sample volume can be achieved by using non-contact, mechanical or chemical stimulations (Capiau et al. 2016).

There are commercially available sampling or diagnostic kits designed for tear sampling (Posa et al. 2013; Sambursky et al. 2013), such as AdenoPlus (Sambursky et al. 2013). AdenoPlus is an integrated sampling and diagnostic tool for adenoviral conjunctivitis (more commonly known as “pink eye”) diagnosis. To perform the test, an examiner needs to swab the interior of patient’s eyelid with the pad on the sample collector (Fig. 17a). After sampling, the sample collector with the tear sample is combined with a test cassette. The absorbent tip of the combined cassette is then placed in a vial of buffer solution. The result is usually ready in 10 min based on the color change of the absorbent material.

**Fig. 18:**
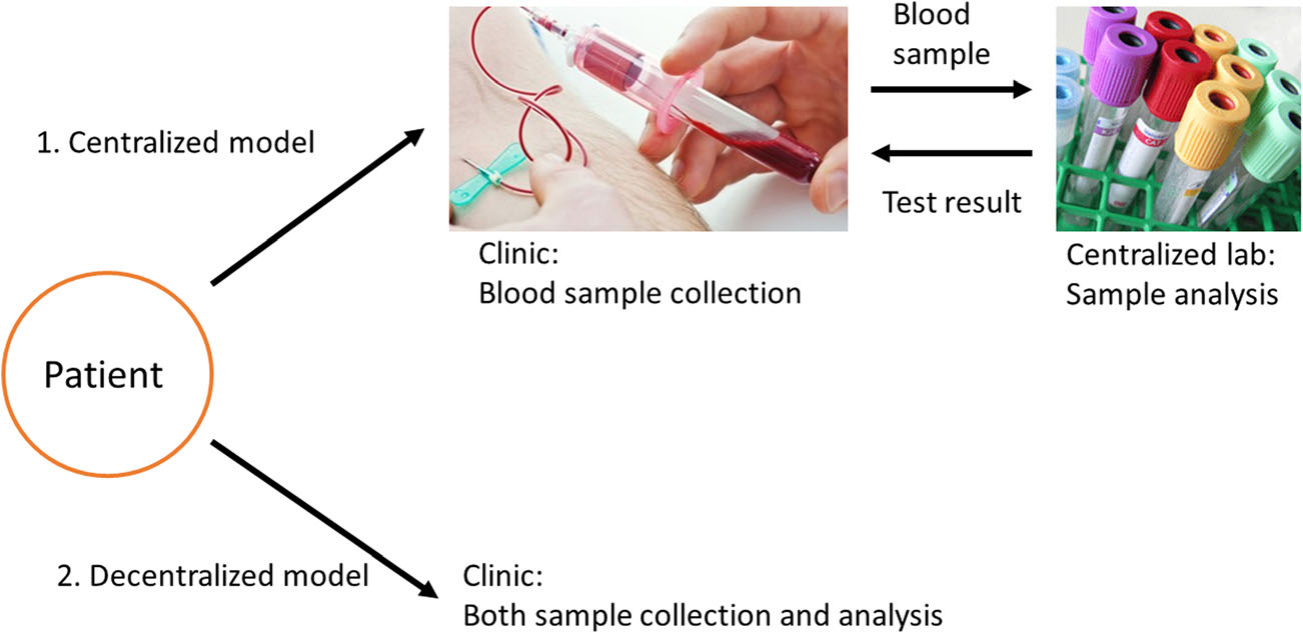
Centralized vs. decentralized diagnostic models. The centralized model involves a centralized laboratory in the diagnostic process and thus lengthens the turnaround time due to sample handling and delivery. In contrast, sample collection and analysis can be done at the same or nearby location under a decentralized model. Pictures retrieved from https://www.butlertech.org/event/phlebotomy-program-starts/ and http://www.johnstoncc.edu/continuing-education/allied-health/phlebotomy/index.aspx

In a clinical study, Sambursky et al. reported comparing the sensitivities and specificities between AdenoPlus and the conventional approach (cell culture + immunofluorescence assay) for diagnosing adenoviral conjunctivitis. AdenoPlus delivered a sensitivity of 85% and a specificity of 98% (Fig. 17b). Both values were closed to the conventional approach (85% and 99%). The simple setup and low requirement on training hugely facilitated the diagnosis of adenoviral conjunctivitis. More importantly, the test only takes about 10 min. These advantages are very critical for the management of the infectious disease.

## Microsampling: Its role in the society beyond science

### Drivers of the microsampling field

Aside from the improvement in molecular tests, the miniaturization trend in the sampling field is largely driven by the evolving micro-manufacturing techniques and their increasing availability. For example, benchtop 3D printers, such as MiiCraft+, usually cost below U$5000 with a resolution up to 40 μm(Miicraft+ Tech Specs n.d.). A print job usually costs less than a dollar on average. The availability of these low-cost manufacturing techniques hugely facilitates the creation of device prototypes and new diagnostic platforms.

On a social level, the trend in the sampling field aligns with the demand for a patient-centric diagnostic experience (Graber et al. 2017; Lee et al. 2017). With a growing concern on the patient’s individual feelings, patients desire for cheaper, more convenient and less invasive diagnostic procedures (Graber et al. 2017), microsampling could be the key in this transforming diagnostic market.

### The business aspect of the diagnostic world

#### Business model of current diagnostic market

The business model of diagnostic services is also experiencing drastic transformation. The current centralized testing system mainly involves hospitals/clinics outsourcing their clinical services to a central diagnostic laboratory (Tsai et al. n.d.). As the maintenance cost of a diagnostic laboratory is high (especially for smaller clinics), most hospitals do not maintain their own laboratories except for some services that require immediate turnaround, such as an intensive care unit. Centralized testing is often considered a more economical model as testing laboratories can leverage the economies of scale by serving multiple clients, and hospitals can use their diagnostic services at a lower price.

Under a centralized model, the diagnosis-to-treatment process, however, is often considered time-consuming from a patient’s perspective. The transport of samples/results can make it difficult to have a treatment prescribed for a patient in one doctor visit (Fig. 18). Oftentimes, the entire diagnostic process can take from hours to weeks depending on the nature of the test. For example, Cohen et al. reported the total diagnostic times (from sample collection to receipt of results) of pulmonary tuberculosis (TB) in South Africa with two different diagnostic tests; acid fast bacilli (AFB) and Xpert MTB/RIF assay (Cohen et al. 2014) (Xpert). While AFB is a technique that is readily available in most clinics, Xpert offers a higher test sensitivity but is only offered in centralized diagnostic laboratories due to its higher operational cost. However, Xpert results took twice as long as AFB results to reach clinicians (6.4 vs 3.3 days). In some cases, AFB could provide sufficient medical information for making a clinical decision. This suggested that the use of centralized test (Xpert) might lead to diagnostic and treatment delays, and the two tests should be combined to achieve the optimal diagnostic outcome. It is not ideal to have a diagnostic time delay in urgent disease conditions as there is a potential for progression of the condition by the time the patient receives treatment. Therefore, decentralizing the current diagnostic business model may reduce the test turnaround time and improve disease management outcome.

**Fig. 19:**
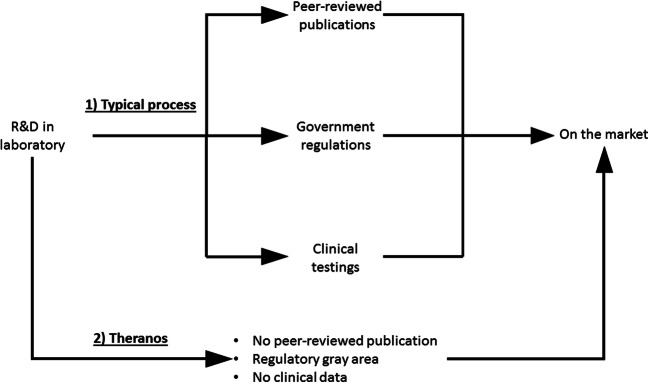
Typical process vs. Theranos’ product development process. Theranos moved their diagnostic platform directly from R&D stage to the market. It skipped the peer review, clinical testing and government clearance procedure

#### Benefits of a decentralized model and how microsampling is driving the evolution of the diagnostic field

As opposed to a centralized model, a decentralized model involves individual hospitals or departments managing diagnostic services by themselves. This can be beneficial in numerous aspects. Primarily, diagnostic procedures can be more accessible to patients. As well as a reduction in test turnaround time as there isn’t a third party involved. The clinical decisions can also be made in a timely manner. Finally, an effective scaling-up can be achieved when relevant POC devices are available. All these benefits can potentially improve treatment outcomes, especially for patients who are in remote areas or require regular diagnostic services (Kip et al. 2017; Ziemniak et al. 2011; Dincer et al. 2017).

The decentralization of diagnostic services is closely related to microsampling as most POC devices require only a small amount of sample. There are some POC devices on the market that can provide immediate diagnosis at the bedside or at the clinic. For instance, i-STAT (Abbott) is a blood analyzer designed to be used in hospitals, especially the emergency department, for doctors to instantly analyze patient’s a blood sample and provide clinical decision. The device can detect up to 28 biomarkers, including blood gases and cardiac markers, with a relatively small sample (<100 μL) when compared to conventional approaches (>0.1–1 mL).

The platform consists of a handheld device and a disposable cartridge. Once the blood sample is drawn from the patient, two to three drops of the patient’s blood sample can be inserted into the cartridge. The cartridge with the blood sample can then be inserted into the handheld device for analysis. The platform only requires <6 min from blood draw to results as opposed to >2 h for a centralized laboratory (i-STAT Handheld Blood Analyzer | Abbott Point of Care n.d.). This allows patient care or any follow-up medical procedures to be delivered in a timely manner.

Although POC platforms like i-STAT are relatively easy to use, the majority of existing POC platforms are designed to be operated by professionals (Dincer et al. 2017). With the improvement in microsampling, diagnostic tests can potentially become even simpler and widespread. The simplification and decentralization of diagnosis may also reduce the workload of highly trained medical personnel. As some of the simplified diagnostic procedures require less training and can be performed by general technicians or even patients themselves, specialists can delegate some of their work and focus on the most important tasks that only they can do, such as discussing important medical decisions with patients. The tape stripping biopsy from DermTech is a good example of such an idea. The tape stripping biopsy does not require a dermatologist to perform so can significantly save their time as compared to conventional approaches.

#### Impacts of a decentralized business model on other stakeholders

Beyond from the obvious benefits on patients and medical professionals, it is crucial to consider other key stakeholders involved in a decentralized business model, such as policy makers and insurers. Policy makers play an essential role in facilitating the availability of high quality POC devices in the market. However, existing guidelines and policies may not provide clear recommendations on how to include POC platforms in hospitals, clinics or patient’s home (Pai et al. 2012). One of the main reasons is the field is rapidly evolving, and some technologies do not have strong clinical data to support the commercialization process. Policy makers can only provide positive recommendations when there is a strong evidence on the feasibility and safety of POC platforms.

In an ideal scenario, POC platforms should offer quick and easy disease screening at an early stage. This will potentially lead to reduced spending on expensive diagnostic equipment or treatment costs after the condition progresses (Asha et al. 2014). As a result, this may avoid a significant amount of wasted medical resources, and the government spending on healthcare can be better allocated to other areas in need.

The impact of a decentralized business model on insurers is unclear. However, their influence in driving microsampling and POC platforms should not be ignored. While POC platforms may effectively reduce later stage treatment and diagnostic costs, an increasing consumption of POC tests might represent an increase in reimbursement costs (Schnippel et al. 2012), such as for the case of Xpert for HIV diagnosis in South Africa (Cohen et al. 2014; Schnippel et al. 2012). The use of Xpert instead of conventional central lab approach resulted in a 51% increase in point-of-treatment cost. Depending on how the healthcare reimbursement system is being structured, the financial impact on the insurer cannot be easily determined. However, if insurers do not support the reimbursement of a POC platform, this may prevent the uptake of the platform by the market. Patients and clinics may choose conventional lab testing services over POC tests as most POC tests are more expensive (Schnippel et al. 2012).

### Challenges in microsampling

One of the main challenges in microsampling is the protocol standardization. Having a standardized testing protocol may be challenging when patients are involved in the sampling procedure. Subtle differences between protocols may introduce variations and impact the reliability of the test, especially for ones that require highly accurate or quantitative results. Thus, some diagnostic procedures that are related to important medical decisions should ideally be performed by trained professionals.

For instance, the inappropriate performance of tape stripping biopsy from DermTech for melanoma diagnosis by patients themselves instead of trained professionals might lead to misdiagnosis and potential disease progression. Even with detailed instructions, results from medical professionals presented lower test specificity before training (Gerami et al. 2017) (53% vs 69%). This indicated the importance of protocol standardization and training. Therefore, there should be a balance between decentralization and test reliability depending on the nature of the diagnostic test. A moderate level of decentralization is desired in order to achieve an optimal disease monitoring outcome.

Besides, not all diagnostic tests can be or should be miniaturized. For instance, an overly small amount of blood sample may not contain sufficient biological information for analysis. While a drop of blood from a finger-prick is usually <25 μL (Waltz 2017), most commercially available blood testing devices require a minimum amount of 100 μL to provide a reliable result (Waltz 2017; Kip et al. 2017). This sample demand is largely due to the limited number of detectable analytes in the blood sample. Some biomarkers are not abundant in the bloodstream. The probability of containing a detectable level of biomarkers in a small drop of blood is low. If there are none/not enough biomarkers to be detected and amplified in a sample, the potential for patients receiving an inaccurate test result exists.

The uniformity of biomarkers in our body should be taken into consideration as well. Small molecules, such as glucose, can move easily between different body compartments, and the concentrations of these small molecules are relatively uniform throughout the body (Waltz 2017). In contrast, larger molecules, such as protein, may have a different concentration in the fingertip than in the rest of the body. This is also one of the reasons why some popular POC platforms, such as i-STAT, only offer a limited number of tests through finger-prick blood sample.

In fact, there are only a few scenarios that a small volume of blood is sufficient for analytical purpose (Waltz 2017; The Saga of Theranos: Crucial Lessons for Clinicians and Pathologists 2016): Firstly, analytes are abundant in the bloodstream, such as drugs and many viruses; Secondly, tests rely mainly on qualitative instead of quantitative analysis (e.g. presence/absence of a biomarker); Finally, only the isolation of genetic materials is needed (e.g. sequencing). Any applications other than those mentioned above continue to be a challenge.

The impact of sample handling and logistical burden is often overlooked. Some of the blood sampling techniques, such as DBS and VAMS, require 8–24 h of drying time prior to sample delivery and analysis (Parker and Cubitt, 1999; Kok and Fillet 2018). These requirements increase the risk of sample contamination and jeopardize the test accuracy. For such a reason, reported success rates of DBS ranged between 30 and 60% under resource-constrained settings (Ziemniak et al. 2011; Williams and McDade 2009). Clearly, a technological gap exists between handling and logistics that needs to be addressed. Microsampling-based diagnostic platforms that can provide reliable and low-cost disease diagnosis are desired.

### The commercialization of a microsampling platform

For a new microsampling technique to reach the commercialization stage and eventually enter the market is a long haul. Simply by addressing the above obstacles and creating a functional sampling/diagnostic device in the laboratory is merely a starting point. Before entering the market, a new device must go through a high level of scrutiny from three parties; the regulatory body, the scientific community and the public. To demonstrate the importance of these three parties, Theranos is one of the best negative examples in recent years.

Theranos’ CEO Elisabeth Holmes founded the blood-testing company in 2003 at the age of 19 (Elizabeth Holmes, Theranos CEO, Is Charged With Fraud | Fortune n.d.). In contrast to conventional blood sampling approaches, Holmes claimed that her company could run more than 200 diagnostic tests faster and cheaper than existing approaches with only a few drops of finger-prick blood (<100 μL) (The Saga of Theranos: Crucial Lessons for Clinicians and Pathologists 2016). The amount of sample combined with the number of tests is unrealistic even with today’s standard in 2018 (Waltz 2017).

Although Theranos had successfully entered the market through the partnership with Walgreens in 2013, there was a complete lack of transparency around Theranos’ groundbreaking diagnostic platform (The Saga of Theranos: Crucial Lessons for Clinicians and Pathologists 2016). On one hand, the science behind Theranos’s technology was unclear to the scientific community. The company had never published any peer-reviewed journal or relevant clinical data on their signature diagnostic platform. On the other hand, the company bypassed FDA’s regulatory clearance by claiming that their diagnostic platform was designed and built inhouse (Waltz 2017). In the US, most centralized diagnostic laboratories are required to go through the FDA for devices purchase from other manufacturers before providing diagnostic services (Waltz 2017; The Saga of Theranos: Crucial Lessons for Clinicians and Pathologists 2016). Theranos was clearly leveraging this grey area to survive.

In late 2015, the Wall Street Journal revealed that the company delivered a series of inaccurate test results. The company simply diluted patient’s samples to run the majority of their tests with conventional blood testing devices inappropriately (Waltz 2017). The Centers for Medicare and Medicaid Services (CMS) also criticized in a public letter that Theranos’ tests ‘posed immediate jeopardy to patient health and safety’ (Waltz 2017).

The CEO of Theranos, Elizabeth Holmes, had also been banned from running a laboratory for at least two years (Bad Blood: The Decline And Fall Of Elizabeth Holmes And Theranos n.d.). Since then, Theranos has laid off hundreds of its employees and now faces multiple lawsuits. Fast forward to the present, Theranos is shifting their focus on a new diagnostic platform, however, is still struggling to recover. Whether the public will still put their trust on the same company that jeopardized their health is questionable. In June 2018, Elizabeth Holmes was charged with fraud and has resigned as Theranos’ CEO (Elizabeth Holmes, Theranos CEO, Resigns, Is Charged With Fraud | Fortune n.d.).

There are a few lessons we can learn from Theranos’ story. Firstly, the peer review process of the scientific community is where the replicability and feasibility of a technology can be properly examined and should not be omitted. Therefore, having a certain amount of publications is an ideal way to demonstrate the science behind a new device. Secondly, the regulatory body is also responsible for ensuring the safety of diagnostic devices entering the market. However, any change in the regulatory system should maintain a balance between patient safety and innovation. An extreme regulation may stifle creativity in the field. Finally, any novel diagnostic devices should obtain sufficient amount of clinical data in order provide transparency to the public (Fig 19).

### The future of diagnostic field

While Theranos was unsuccessful in the diagnostic field, the hasty acceptance of its technology by the market has validated the demand for microsampling-based diagnostic platforms. There is obviously a need for low-cost, simple and easily accessible pathological services, especially in the blood area.

Nevertheless, the damage being done by Theranos to the field can be long-lasting. Any companies of a similar nature would likely encounter more difficulties in raising capital as investors are becoming more cautious. However, this will encourage biotech companies and researchers to gain credibility for their studies through scientific and regulatory frameworks in order to convince investors and consumers. It is likely to slow down the product development process with the heightened level of scrutiny, but companies that can withstand the scrutiny will end up producing much safer and far more reliable diagnostic platforms.
